# Mitochondria-lysosome coupling contributes to lysosome acidification and aging

**DOI:** 10.1016/j.molcel.2026.05.004

**Published:** 2026-05-29

**Authors:** Qingqing Liu, Seungmin Yoo, Zhixin A. Zhang, Liying Li, Hetian Su, Lingraj Vannur, Alexandra C. Wooldredge, Jun-Wei B. Hughes, Pierre-Yves Desprez, Nan Hao, Gordon Lithgow, Julie K. Andersen, Malene Hansen, Judith Campisi, Chuankai Zhou

**Affiliations:** 1 Buck Institute for Research on Aging, 8001 Redwood Blvd., Novato, CA 94945, USA; 2 Department of Molecular Biology, University of California, San Diego, La Jolla, CA 92093, USA; 3 Lead contact

## Abstract

Nearly all cellular processes are pH dependent. The acidic pH inside the lysosome (vacuole in yeast) is essential for cellular content degradation, signaling, and autophagy. Defects in lysosome/vacuole acidification are a conserved hallmark of aging and age-related diseases. Traditionally, the lysosome/vacuole is thought to import free protons (H^+^) from the surrounding neutral cytosol. Here, we uncovered a conserved lysosome/vacuole acidification mechanism from yeast to human involving lysosomal/vacuolar uptake of H^+^ pumped out by mitochondrial electron transport chain through mitochondria-lysosomes/vacuoles membrane contacts. Aging/senescence-associated disruption of mitochondria-lysosome/vacuole contacts causes lysosomal/vacuolar de-acidification, which can be reversed by either expressing an engineered linker to connect these two organelles or through an asymmetry-dependent rejuvenation process in daughter cells. Preserving lysosomal acidification in senescent human cells prevents the induction of major senescence-associated secretory phenotype factors and restores autophagic flux. These findings reshape our current understanding of the mechanisms underlying lysosomal/vacuolar (de-)acidification in both young and aged/senescent cells.

## In brief

While classical models propose that lysosomes/vacuoles are acidified using free cytosolic protons, Liu et al. reveal that mitochondrial electron transport provides a substantial proton source through Mito-Lyso/Vac membrane contacts. Loss of these contact sites during yeast replicative aging and human cellular senescence drives lysosomal/vacuolar dysfunction and induces SASP factors.

## Graphical abstract

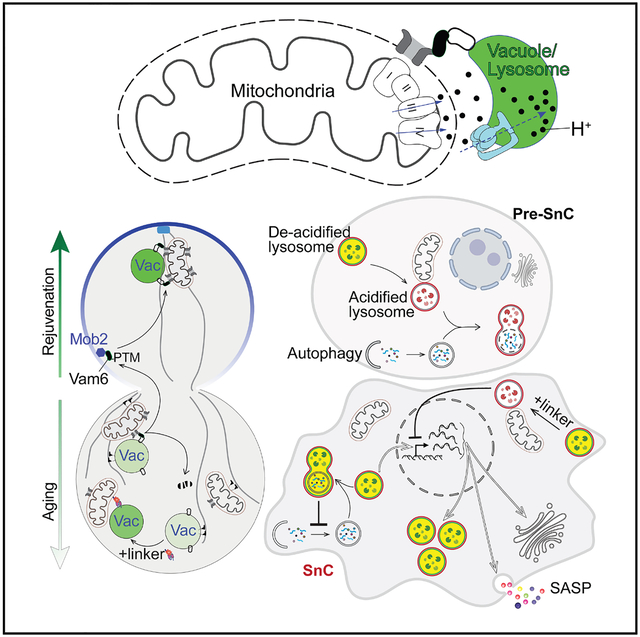

## INTRODUCTION

Eukaryotic cells compartmentalize their contents and functions into various membrane-bound organelles, each providing a specialized environment enriched with specific molecules to support optimal enzyme activity and metabolic processes. Proton concentration, reflected by the pH of each individual organelle, plays a critical role in this context, since protonation determines the folding, structure, interaction, and catalytic reaction of proteins and RNAs as well as the reactivity of metabolites.^1^ For example, the lumen of the lysosome (vacuole in yeast) normally maintains an acidic pH, critical for the activity of various catabolic enzymes that degrade cellular contents in order to maintain cellular proteostasis.^2–4^ It is commonly believed that lysosomes/vacuoles use the conserved V-ATPase complex to import free H^+^ from the surrounding pH-neutral cytosol to acidify their lumen to as low as pH 4.5.^3–6^ Cytosolic metabolism, including glycolysis, is an important source of cytosolic H^+^ by producing free H^+^ and generating intermediates that disassociate into charged forms and H^+^ at physiological pH.^7^ In addition, the catabolic processing within the mitochondrial matrix generates CO_2_ (in the form of carbonic acid, H_2_CO_3_) and H^+^, which must be exported to maintain mitochondrial pH homeostasis.^8^ However, the estimated number of free H^+^ in the cytosol of a single yeast cell (which has a volume of ~48 μm^3^ with a pH of 7 or higher) is <3,000.^7,9,10^ Cytosolic metabolites (e.g., inorganic phosphate) and proteins, which have multiple protonatable groups, have strong affinity for these <3,000 free H^+^. These cytosolic buffering molecules are 5–10 orders of magnitude more abundant than cytosolic free H^+^ levels and their affinity for free H^+^ significantly reduces the availability and diffusion of H^+^ across the cytosol.^11,12^ As a result, the lysosomal/vacuolar V-ATPase must compete with large numbers of cytosolic molecules for free H^+^ and may only be capable of capturing H^+^ generated in its immediate vicinity.

In addition, previous studies underscore a remarkable heterogeneity in lysosome pH, even though lysosomes are subject to rapid transportation and mixing within human cells.^13,14^ Ammonia (NH_3_), an endogenous metabolite, can act as a lysosomotropic weak base, disrupting lysosomal acidity.^15^ When lysosomes are transiently neutralized with NH_4_Cl, those located in the perinuclear region re-acidify significantly faster than those in peripheral areas.^14^ Similarly, the asymmetric cell division in budding yeast, which produces an aging mother cell and a stem-cell-like daughter cell (bud) that resets its aging clock (rejuvenation), creates a significant pH difference between the vacuoles of the mother and daughter cells, despite sharing the same neutral cytosol and the fact that the vacuoles in the daughter cell are inherited from the aging mother cell.^16^ In this case, the vacuoles inside the aging mother cells undergo age-dependent loss of acidification, which is conserved in metazoans, while such alkalized vacuoles, when inherited by the rejuvenated daughter cell, undergo re-acidification.^16^ It remains unclear how ubiquitously distributed proton sources across the cytosol could give rise to spatial difference in lysosomal/vacuolar pH and what is the underlying cause of this uneven acidification of lysosomes/vacuoles.

In this study, we uncovered a conserved mechanism in which protons pumped out of the mitochondria by the electron transport chain (ETC) contribute to vacuole/lysosome acidification through membrane contacts between these organelles. In both yeast and human cells, loss of these contacts during aging or senescence contributes to vacuolar/lysosomal de-acidification, whereas restoring contact with an engineered linker improves acidification. In yeast, this mechanism also contributes to vacuole re-acidification during daughter cell rejuvenation. Together, these results identify mitochondria-contact-dependent proton transfer as a conserved component of vacuole/lysosome acidification.

## RESULTS

### Changing mitochondrial function influences vacuolar acidification

**Figure 1.**
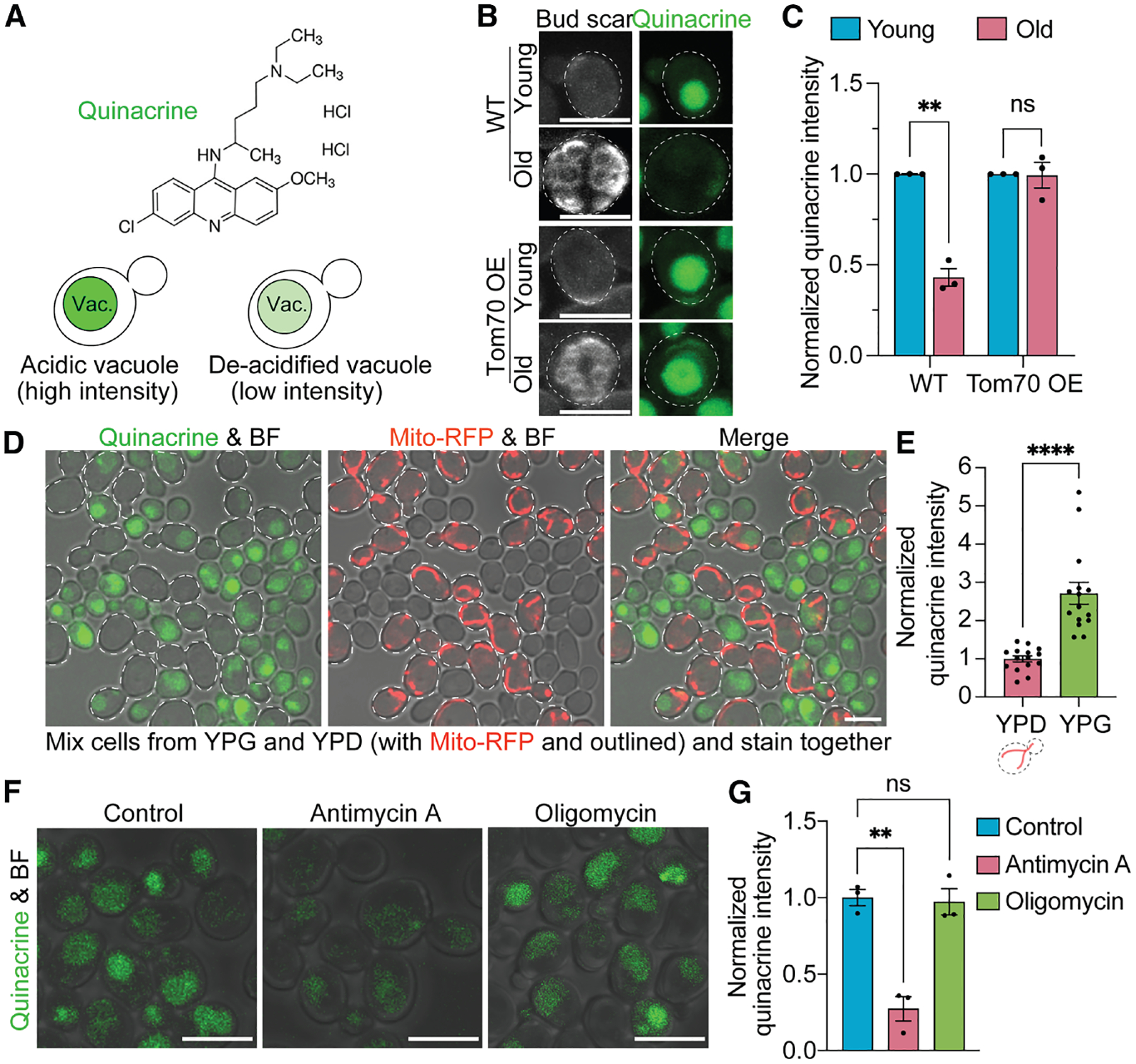
Changing mitochondrial function influences vacuolar acidification (A–C) Representative images and quantification of quinacrine staining in young and old cells of wild-type (WT) and Tom70 overexpression (Tom70 OE). Quinacrine intensity is normalized to young cells in each group (same in other figures). (D and E) Representative images and quantification of quinacrine staining of cells cultured in YPD and YPG media. To avoid quinacrine staining variation, cells from YPD and YPG media were mixed in a 1:1 ratio and stained together (same in other figures). Cells from YPD (dashed line) contain mito-RFP as a marker. BF, bright field. (F and G) Representative images and quantification of quinacrine staining of cells cultured in YPD and treated with antimycin A (ETC inhibitor) or oligomycin (F0-F1 ATPase inhibitor). Bar graphs are means ± standard error of mean (SEM, see STAR methods for statistics, same for other figures). Scale bars: 5 μm.

Using a well-established fluorescent probe for vacuole acidification,^16,17^ we serendipitously discovered that overexpression of the mitochondrial import receptor protein TOM70 (Tom70 OE) from different promoters, which preserves mitochondrial membrane potential (MMP) during aging,^18^ prevented age-associated vacuole de-acidification in yeast (Figures 1A–1C and S1A–S1D). In a similar vein, certain growth conditions that enhance the MMP including caloric restriction and a nonfermentative carbon source (glycerol) also intensified vacuole acidification compared with cells grown in a regular 2% glucose medium (Figures 1D, 1E, S1E, and S1F).^16^ The MMP is generated by the proton pumps of ETC complexes, which pump protons out of mitochondrial matrix into the intermembrane space to build an electrochemical proton gradient across the inner mitochondrial membrane. Unlike the inner mitochondrial membrane, which is impermeable except through specific transporters, the mitochondrial outer membrane permits non-specific permeation of molecules up to 5 kDa.^7,19^ As protons in the intermembrane space are free to diffuse in all directions, the porous nature of the mitochondrial outer membrane may facilitate the diffusion of some protons out of the mitochondria that could potentially drive vacuolar acidification. Indeed, blocking the ETC complex with antimycin A, which specifically inhibits complex III, significantly compromised the acidification of the vacuole (Figures 1F and 1G). The MMP/H^+^ gradient is also critical for producing ATP via the F1-F0 ATPase. However, inhibiting the F1-F0 ATPase with oligomycin did not affect vacuolar acidification (Figures 1F and 1G). This is likely because ATP generated via cytosolic glycolysis is sufficient to power V-ATPase in yeast cells grown in glucose-containing medium.^20,21^ Although the vacuolar V-ATPase can uptake free protons from the cytosol, these data suggest that the mitochondrial MMP/H^+^ gradient is an important source of H^+^ for vacuolar acidification. The contribution of the mitochondrial MMP/H^+^ gradient to vacuole acidification is likely not exclusive, as previous studies in human cells have shown that increased mitochondrial MMP can cause cytosolic acidification as well.^19,22,23^

### Mitochondria-vacuole contacts are critical for vacuolar acidification

**Figure 2.**
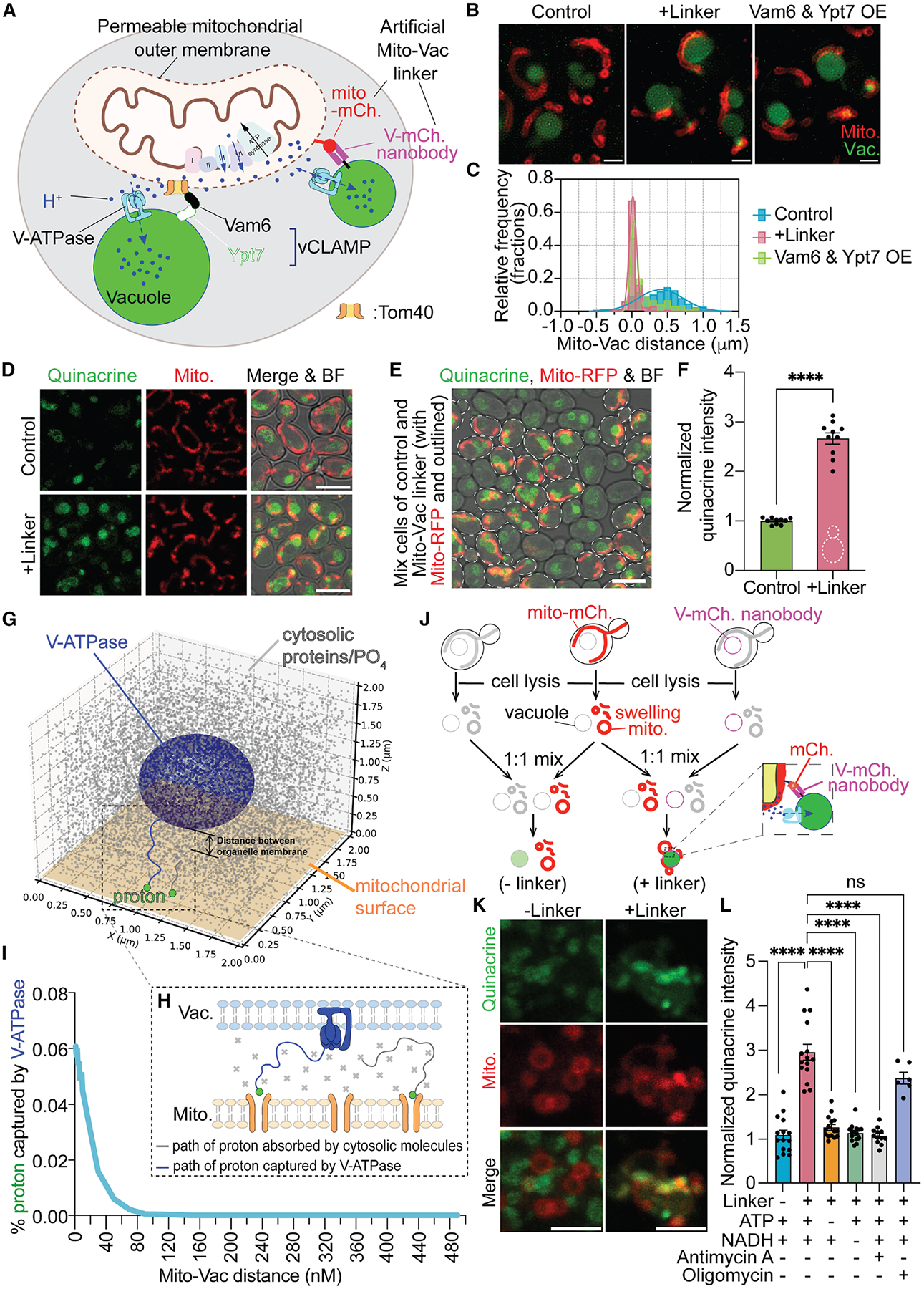
Mitochondria-vacuole contact is critical for vacuolar acidification (A) Model and schematic diagram for the mitochondria-vacuole interaction and proton flow. mito-mCh., mCherry-Fis1tm labels mitochondria; V-mCh. nanobody, VPS10-mCh nanobody is anchored on vacuole surface. (B and C) Representative super resolution images and quantification showing that expressing Mito-Vac linker brings mitochondria and vacuole together. The pulse expression of the Vps10-mCh nanobody was done with 30 min galactose induction. Mito, mitochondria, shown by mCh-Fis1tm; Vac, vacuole. Scale bar, 1 μm. (D–F) Representative images and quantification of quinacrine staining in control and Mito-Vac-linker-expressing cells stained in separate reactions (D) or stained simultaneously in the same reaction (E). BF, bright field. (G–I) Molecular simulation of proton transfer between organelles. (J–L) *In vitro* assay to detect the transfer of proton from mitochondria to vacuole. See details in STAR Methods. Scale bars, 5 μm if not mentioned.

The cytosol is densely populated with buffering molecules (for example, phosphate, PO_4_^3−^) exceeding 50 mM and a myriad of amino acid side chains of proteins with a strong affinity for free H^+^.^7,12^ Thus, it is possible that most of the H^+^ released from the mitochondrial outer membrane are absorbed by cytosolic buffering molecules before they can reach the V-ATPase. This prompted us to consider the possibility of inter-organelle membrane contact sites between the mitochondria and the vacuole (Mito-Vac contacts) acting as conduits for the transfer of protons from the mitochondria to the vacuoles, thereby facilitating their acidification. Previous studies from different labs have identified conserved proteins involved in mediating Mito-Vac contacts. In yeast, the vacuole membrane-anchored Rab GTPase Ypt7 and its effector Vam6 form a protein bridge with Tom40, the import channel for the TOM complex located in the outer mitochondrial membrane (Ypt7-Vam6-Tom40 bridge was named vCLAMP, Figure 2A).^24–27^ These organellar contacts are conserved in mammalian cells, where RAB7 (homolog of Ypt7) regulates mitochondria-lysosome contact formation.^28,29^ It has been shown that the knockdown of Vam6 reduces, while its OE augments, Mito-Vac contact in yeast.^26^ Consistent with the hypothesis that mitochondria could contribute to the acidification of vacuoles via Mito-Vac contacts, we observed a significant enhancement of vacuolar acidification in yeast cells that overexpressed Vam6 or Ypt7, while deleting these proteins significantly impaired vacuolar acidification (Figures S2A–S2G). This effect of Mito-Vac contacts on vacuolar acidification was further confirmed with a ratiometric pH-sensitive fluorescence sensor (Figures S2H–S2J).

Given the multifaceted functions of vCLAMP proteins within the cell,^30,31^ we further investigated the role of Mito-Vac contacts in facilitating H^+^ transduction from mitochondria to vacuoles by using alternative approaches to exclude indirect effects arising from manipulating Vam6 and Ypt7. To this end, we developed an engineered linker (Mito-Vac linker) devoid of any other cellular functions. We fused mCherry (mCh) to the transmembrane domain of the mitochondrial outer membrane protein Fis1 (Fis1tm), facilitating the insertion of mCh into the mitochondrial outer membrane (mito-mCh).^32^ To bridge vacuoles with mitochondria, we induced a pulse expression of a mCh-specific nanobody^33^ fused to Vps10 which drives the localization of Vps10-mCh nanobody to the vacuole membrane (V-mCh nanobody), where it can interact with the mito-mCh on the mitochondrial membrane (Figure 2A). We first confirmed that transient expression of the V-mCh nanobody increases mitochondrialvacuole contacts to a similar extent as OE of the endogenous vCLAMP tethering proteins Vam6 and Ypt7 (Figures 2B and 2C). Consistent with our observations from endogenous vCLAMP OE, increasing Mito-Vac contacts with this engineered linker enhanced vacuolar acidification, whether quinacrine staining was performed on control and linker-expressing cells in separate reactions (Figure 2D) or simultaneously to minimize batch-to-batch variation (Figures 2E and 2F). This conclusion is further supported by the recently developed ratiometric vacuolar pH sensor v-SEP,^34^ which likewise showed that Mito-Vac linker expression enhances vacuolar acidification (Figures S2K and S2L). This enhanced Mito-Vac contact and vacuolar acidification was not due to Vps10 expression and was specific to the Mito-Vac linker, as neither Vps10 alone nor an endoplasmic reticulum (ER)-Vac linker showed this effect (Figures S2M–S2R). Moreover, consistent with the role of vacuolar acidification in autophagy, expressing this Mito-Vac linker also led to a faster turnover of green fluorescent protein (GFP)-Atg8 puncta (representing autophagosomes), indicating increased autophagic flux (Figures S2S and S2T).^35^

To further test the hypothesis that membrane contacts between these two organelles serve as conduits for proton transfer, we measured the pH of the intermembrane space between the vacuole and mitochondria in yeast. We used a genetically encoded ratiometric pH biosensor based on a tandem heterodimer of pH-sensitive superfolder GFP (sfGFP) and relatively pH-insensitive mCh. We validated the pH sensitivity of this biosensor by incubating yeast cells expressing the heterodimer in the cytosol or on the mitochondrial outer membrane with buffers of known pH values, along with the protonophore nigericin, which equilibrates intracellular compartments with the extracellular pH (Figures S2U–S2W). We observed no significant pH difference between the mitochondrial surface and the cytosol in wild-type cells (Figure S2X). This is likely because protons released from mitochondria are rapidly diffused or absorbed by cytosolic molecules outside of the Mito-Vac contact sites, coupled with the fact that in wild-type cells the sfGFP-mCh heterodimer is evenly distributed across the mitochondrial surface—mostly outside organellar contact sites—resulting in an average sfGFP/mCh ratio similar to that of the bulk cytosol (Figure S2Y, left). If these membrane contact sites indeed serve as conduits for proton transfer by excluding cytosolic proton competitors, then recruiting and enriching the pH biosensor at the contact sites should yield a significant signal change (Figure S2Y, right). Consistent with this prediction, when we pulsed the expression of V-mCh nanobody, which binds mCh and concentrates the sfGFP-mCh heterodimer at the Mito-Vac contact site, the sfGFP/mCh ratio significantly decreased (Figure S2Z).

To gain mechanistic insight into the role of membrane contact sites in proton transfer, we carried out *in silico* molecular simulations of proton diffusion and capture, parameterized using experimentally measured physiological values (Figure 2G). In this model, protons were randomly released from the mitochondrial outer membrane and diffused within a cytosolic environment containing proteins and phosphate molecules—both acting as competitive proton sinks—alongside vacuolar V-ATPase complexes positioned at a defined surface density (Figure 2H). The simulations showed that protons released from mitochondria are largely neutralized by cytosolic competitors unless the vacuole membrane is in immediate proximity to the mitochondria, where the vacuole membrane-anchored V-ATPases have a chance to capture these protons (Figure 2I). This suggests that spatial proximity provided by organelle contact sites is critical for V-ATPase to capture mitochondrial protons in the context of a protein-dense cytoplasm.

To exclude cytosolic factors and detect the direct transfer of proton from mitochondria to vacuoles, we lysed the cells to remove cytoplasm and suspended the organelles in pH 7.5 buffer, thereby excluding the contribution from cytosolic metabolism and other cytosolic factors in vacuole acidification (Figure 2J). After mixing the mito-mCh-labeled mitochondria and V-mCh nanobody-labeled vacuoles derived from separate strains, we were able to bridge the connection between mitochondria and vacuoles *in vitro* (Figure 2K, +linker). This mitochondria-vacuole connection depended on the presence of mCh and its nanobody (Figure 2K). Compared with vacuoles without mitochondrial association, those attached to mitochondria were significantly acidified in the presence of an ATP regeneration system and NADH (Figures 2K and 2L). The acidification of vacuoles in contact with mitochondria indicates that there were protons leaving the mitochondria and being captured by the V-ATPase to acidify the vacuole. Indeed, removing NADH, which serves as an electron donor for ETC complexes, or adding antimycin A inhibited the vacuolar acidification and abolished the acidification difference between vacuoles with and without mitochondrial association (Figure 2L). These *in vitro* results, along with observations within live yeast cells, support the transfer of protons from mitochondria to vacuoles via Mito-Vac contacts.

### Mitochondria-vacuole contacts decline during aging

Recognizing the effect of Tom70 OE in preserving vacuolar acidification in aged cells and the crucial role of mitochondrial protons in vacuolar acidification, we next asked whether mitochondria contribute to the age-dependent loss of vacuolar acidification in yeast mother cells and the re-acidification of aged vacuole inherited by daughter cells, resulting in a striking asymmetry of vacuole pH across the mitotic axis.^16^

A previous model suggested that changes in vacuole pH arise from competition between the V-ATPase and the plasma-membrane proton pump Pma1, with the latter expelling cytosolic H^+^ out of the yeast cell.^16,36^ In this model, age-dependent accumulation of Pma1 in mother cells, rather than decline in V-ATPase, reduces the cortical H^+^ concentration immediately beneath the plasma membrane in mother cells, while the Pma1-free daughter cells are able to re-acidify/rejuvenate the inherited vacuole.^36,37^

However, our findings revealed that Pma1 asymmetry depended on culture conditions: only cells experiencing high density (optical density 600 [OD_600_] > 2) and nutrient depletion during overnight culture exhibited asymmetric Pma1 localization, whereas cells continuously maintained at a low mid-log density (OD_600_ ~ 0.5) showed equal Pma1 localization between daughter and mother cells (Figures S3A–S3D). Time-lapse imaging of cells from mid-log culture further confirmed the absence of Pma1 asymmetry between mother and daughter cells during replicative aging and purified old mother cells from such low-density cultures likewise showed no plasma-membrane accumulation of Pma1 (Figures S3E and S3F). Notably, despite the lack of Pma1 asymmetry, cells kept at low mid-log density throughout the experiment still showed asymmetry in vacuole pH between mother and daughter cells (Figure S3G), indicating that the distribution of Pma1 does not correlate with the asymmetry in vacuolar acidification across the mitotic axis. However, Pma1 may still further deplete the limited pool of free H^+^ (~3,000) in the cytosol,^36,37^ thereby impeding V-ATPase-dependent H^+^ import from the cytosol and making other sources of H^+^ crucial for vacuolar acidification. Given that Pma1 is not conserved in metazoans, it is likely that the conserved age-associated lysosome/vacuole de-acidification is driven by alternative mechanisms, such as mitochondrial dysfunction or impaired Mito-Vac contacts.

**Figure 3.**
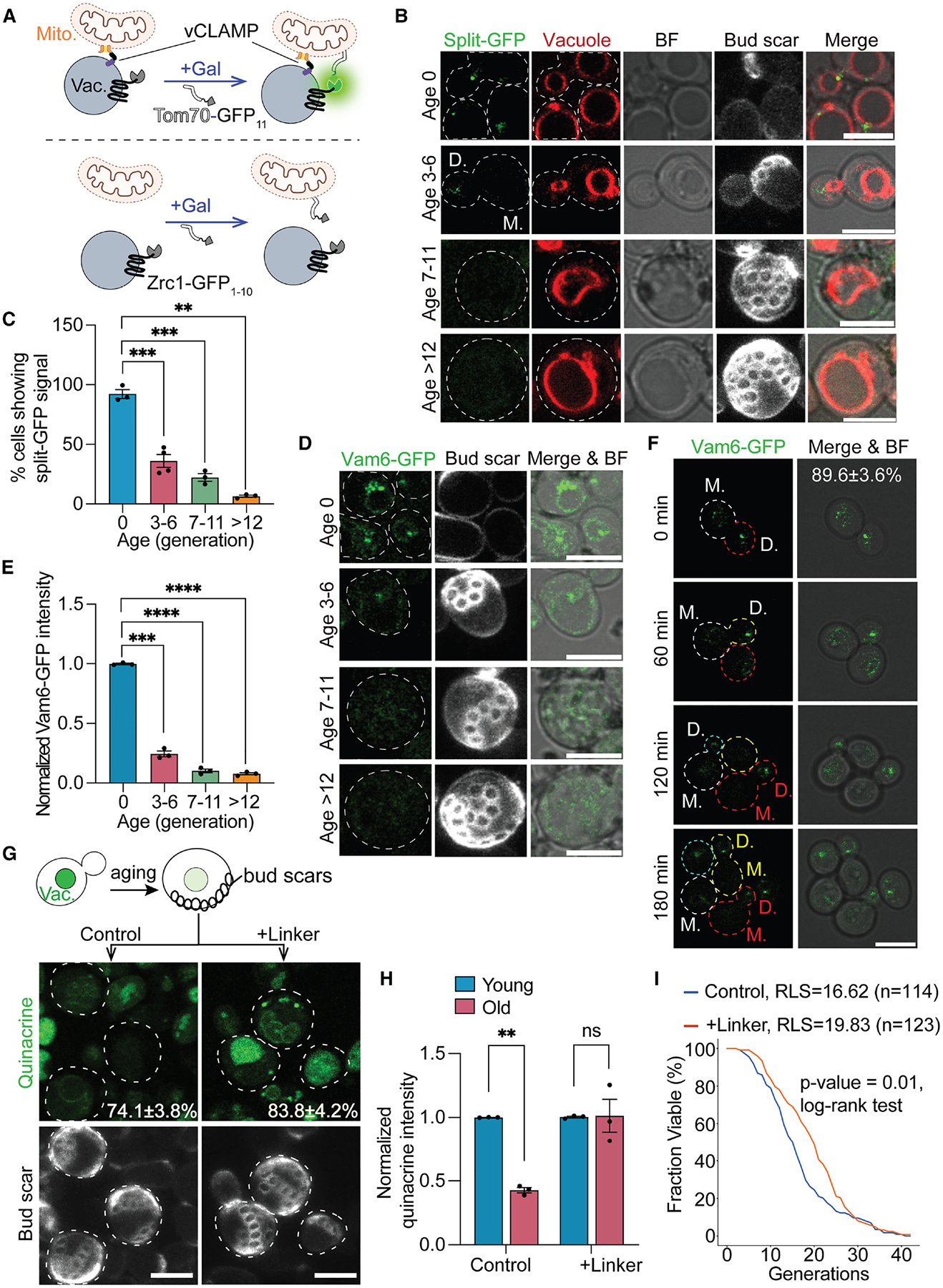
The decline of mitochondria-vacuole contacts during aging (A–C) Schematic illustration, representative images, and quantifications of using split-GFP to visualize Mito-Vac contacts. Cells were initially cultured in YPD until they reached advanced ages and followed by switching to YP-galactose medium to induce Tom70-GFP_11_ expression. BF, bright field; M., mother cell; D., daughter cell. (D and E) Representative images and quantification of Vam6-GFP during aging. (F) Time-lapse images of Vam6-GFP expression level and distribution in mother and daughter/bud cells. Five time-lapse images and 79 budding cells were quantified. M., mother cell; D., daughter cell. The inserted numbers are percentage of cells showing the representative phenotypes (same in other figures). (G and H) Representative images and quantification of quinacrine staining in purified old cells followed by pulse expression of Mito-Vac linker (+linker). (I) Constant low-level expression of Mito-Vac linker extends replicative lifespan. Scale bars, 5 μm.

As vacuole de-acidification occurs early in life and precedes the collapse of the mitochondrial MMP,^16^ we considered the possibility that an age-dependent vacuole alkalization might result from the loss of Mito-Vac contacts between these organelles. We visualized the Mito-Vac contacts between mitochondria and vacuoles using a split-GFP system as in a previous study.^24,38^ The split-GFP system consists of the first ten β strands of GFP (GFP_1–10_) linked to endogenous Zrc1, a low-abundant vacuole membrane protein, while the eleventh β strand (GFP_11_) was fused to pGAL-driven Tom70 on the surface of mitochondria (Figure 3A). A brief induction of Tom70-GFP_11_ allowed reconstitution with vacuole-anchored Zrc1-GFP1–10 when they were within a few nanometers of one another, forming distinct GFP foci that represent Mito-Vac contact sites, as demonstrated previously.^24^ Indeed, the Mito-Vac contact sites reduced significantly during the early stage of aging (Figures 3B and 3C). This decrease was not due to reductions in Zrc1 or pGAL expression used in the split-GFP assay during aging (Figures S3H–S3K). Interestingly, the age-dependent loss of Mito-Vac contact sites in aging mother cells was reversed in daughter cells, aligning with the asymmetric vacuolar acidification across mitosis axis (Figures 3B and 3C, age 3–6 vs. age 0).

This loss of Mito-Vac contacts could at least in part be attributed to the age-dependent reduction of Tom40^39^ and Vam6, two components of the vCLAMP bridge^24^ (Figures 3D, 3E, and S3L). In young cells, Vam6-GFP localizes to the vacuole membrane as foci at the Mito-Vac contact sites.^24,26^ In yeast cells expressing endogenously GFP-tagged Vam6, we found that Vam6-GFP abundance reduced during the early stage of replicative aging and the remaining signal displayed diffuse localization throughout the cytosol without clear enrichment on the vacuole membrane (Figures 3D and 3E). This age-dependent reduction of Vam6 levels was also observed in time-lapse imaging where the mother cells have reduced Vam6 compared with the newborn daughter cells (Figure 3F).

We next investigated whether this age-dependent loss of Mito-Vac contacts could be the primary cause of vacuole de-acidification. If the loss of Mito-Vac contacts is indeed a major driver of age-dependent vacuolar alkalization, then restoring these contacts in aged cells should be able to reverse the loss of vacuole acidification. To test this, we purified replicative old cells in which vacuolar alkalization had already been established. We then transiently expressed the mCh-nanobody-based Mito-Vac linker in these purified old cells. Indeed, transient expression of the Mito-Vac linker in the purified old cells restored their vacuolar acidification (Figures 3G and 3H). This reversibility of old and alkalinized vacuoles upon re-establishment of Mito-Vac contacts suggests that the vacuole and vacuolar V-ATPase remain functional when cells lose vacuolar acidification in early life, although we cannot rule out the possibility that the V-ATPases may eventually lose activity after persistent vacuolar de-acidification in later life. In fact, the abundance of V-ATPase proteins did not decrease during the replicative aging (Figure S3M). Moreover, the constant expression of low levels of this Mito-Vac linker was able to maintain vacuole acidification during aging and significantly extend the replicative lifespan of yeast cells by approximately 20% (Figures 3I and S3N), underscoring the importance of Mito-Vac contacts in preserving proper vacuole function and overall cellular health during aging.

### Mitochondria-vacuole contacts contribute to vacuolar re-acidification in daughter cells

**Figure 4.**
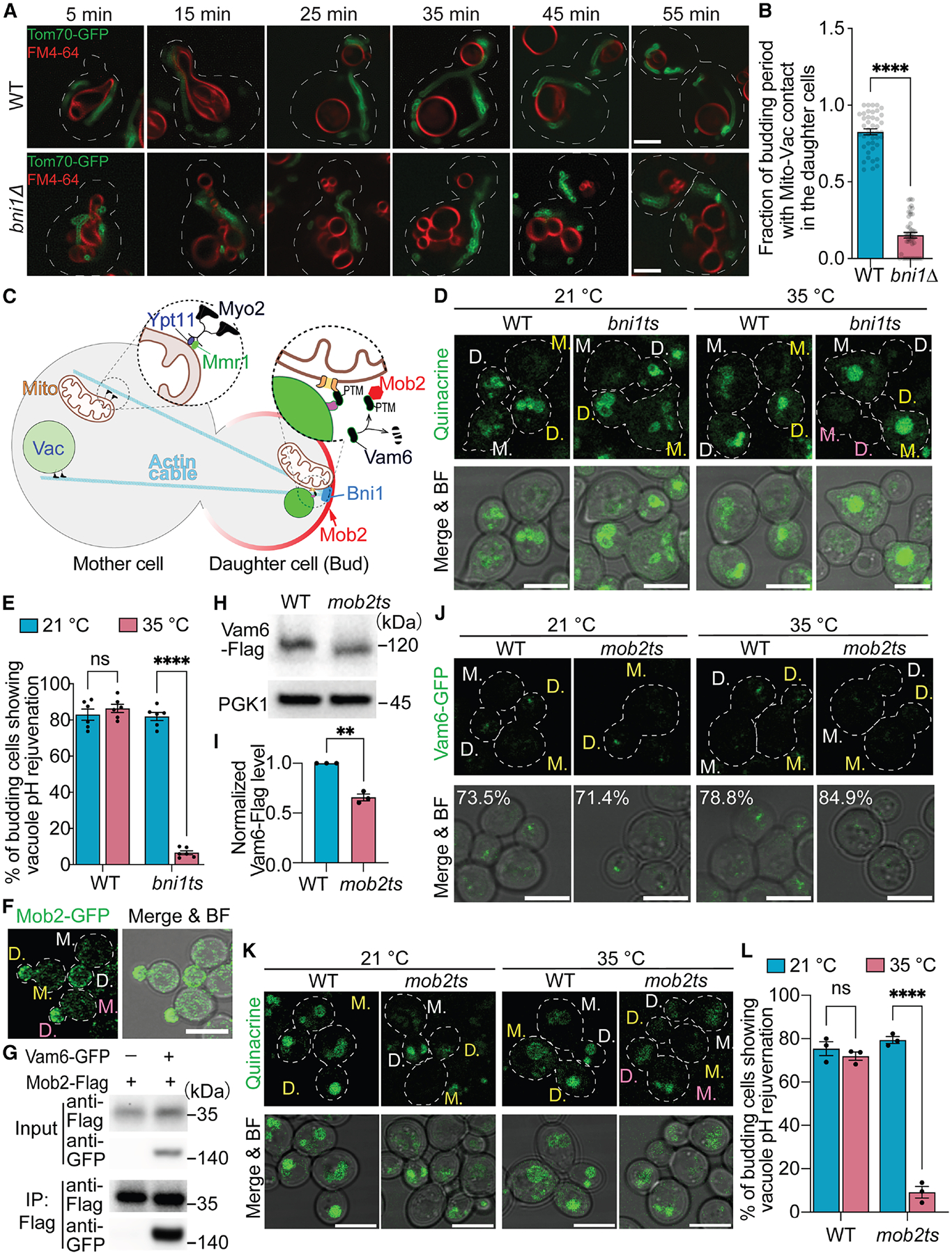
Mitochondria-vacuole contacts contribute to vacuolar re-acidification in daughter cell (A and B) Super-resolution images and quantification of the Mito-Vac contacts in WT and *bni1Δ* cells. Over 40 movies for both WT and *bni1Δ* cells were analyzed to quantify the fraction of the budding period showing Mito-Vac contact. Scale bar, 1 μm. (C) Schematic illustration of organelle inheritance and contact-site regulation during asymmetric cell division. PTM, post-translational modification. (D and E) Representative images and quantification of quinacrine staining in synchronized WT and bni1 temperature-sensitive (*bni1ts*) cells. M., mother cell; D., daughter cell (bud); BF, bright field. (F) Representative image of Mob2-GFP. (G) Representative blot of co-immunoprecipitation (coIP) showing interaction between Vam6 and Mob2. (H and I) Representative immunoblot (H) and quantification (I) of Vam6-FLAG in WT and *mob2ts* cells at restrictive temperature, normalized to PGK1 loading control. (J) Representative images and quantification of Vam6-GFP localization in WT and temperature-sensitive *mob2ts* cells. (K and L) Representative images (K) and quantification (L) of quinacrine staining of vacuole in WT and *mob2ts* cells. Scale bars, 5 μm if not mentioned.

The observed re-acidification of vacuoles in old mother cells upon restoring Mito-Vac contacts is strikingly reminiscent of the rejuvenation of vacuolar acidification observed in daughter cells.^16^ This similarity raises the possibility that an analogous mechanism could contribute to the vacuolar rejuvenation in daughter cells. Indeed, compared with mother cells, the membrane contact between mitochondria and vacuole in budding daughter cells was more than doubled (Figures S4A and S4B). Super-resolution imaging revealed that in wild-type cells, both mitochondria and vacuole migrated from mother cell to the bud tip and form extensive contacts inside the daughter cell during the cell cycle (Figures 4A and 4B). This is consistent with the enhanced Mito-Vac contact observed in daughter cells compared with their aging mother cells (Figures 3B and 3F). This phenomenon is likely because the polarisome, which localizes at the bud tip and contains the bud-enriched formin Bni1, produces polarized actin cables that converge at the bud tip to facilitate the polarized transportation of organelles (Figure 4C).^40,41^ These converged actin cables at the bud tip promote the formation of organelle contacts, a phenomenon previously demonstrated between the mitochondria and ER.^42^

We speculated that a similar process occurs between mitochondria and the vacuole. Indeed, knocking out Bni1, which disrupts actin cable nucleation at the bud tip, not only reduced the membrane contact between mitochondria and vacuole in the bud (Figures 4A and 4B) but also impaired the acidification of vacuole in the daughter cells inheriting both organelles (Figures S4C–S4F). We noticed that the constitutive knockout of *Δbni1* also reduced the vacuolar acidification in the mother cells (Figures S4C and S4D). This may be because the *Δbni1* daughter cells, unable to rejuvenate their vacuoles during the budding stage, mature into mother cells and contribute to the observed phenotype at the population level (Figure S4G). To test this hypothesis, we turned to transient inactivation of Bni1 using a temperature-sensitive mutant (*bni1ts*).^43^ While *bni1ts* cultured at permissive temperature exhibited normal vacuole pH asymmetry, a brief heat shock at a non-permissive temperature nearly completely blocked the acidification of vacuole in the daughter cells (Figures 4D and 4E). The absence of quinacrine staining in daughter cells was not due to a vacuole inheritance defect in *bni1ts* cells, as FM4–64 dye staining indicated the presence of vacuole in the bud, but without accompanying quinacrine staining (Figure S4H). Furthermore, when we depleted the Gal-promoter-driven mitochondria-specific adaptor Mmr1 in the *Δypt11* cells,^42,44–46^ which blocked the polarized transportation of mitochondria to the bud tip, daughter cells also failed to acidify the vacuole inherited from their mother cells (Figures S4I–S4K). Mother cells containing mitochondria also lost vacuolar acidification, likely because the depletion of Mmr1 took 4 h in these mother cells after shifting from galactose to glucose, during which the mother cells gradually lost vacuolar acidification as a result of replicative aging^16^ (Figures S4I, S4J, blue circles, and S4L).

In addition to Bni1 and the converging polarized actin cytoskeleton, we also investigated whether any daughter-specific factors directly regulate vCLAMP components to enhance vacuole acidification in daughter cells (Figures S4M and S4N). Analysis of previous mass spectrometry data revealed that Vam6 interacts with Mob2, a kinase adaptor localized to the cortex of the daughter cell and enriched at the bud tip during polarized growth (Figure 4F).^47,48^ This interaction was confirmed through co-immunoprecipitation (Figure 4G). Knocking down Mob2 using a temperature-sensitive mutant resulted in a shift in Vam6 migration, indicating that Mob2 is necessary for the post-translational modification of Vam6 (Figure 4H). Additionally, Vam6 levels decreased in Mob2 knockdown cells, suggesting that Mob2 is crucial for the stability of Vam6 (Figure 4H and 4I). Since Mob2 is asymmetrically localized in the daughter cell, its role in stabilizing Vam6 aligns with the observed asymmetry in Vam6 abundance between daughter and mother cells, with Vam6 levels being higher in daughter cells compared with their aging mother cells (Figure 3F). Indeed, knocking down Mob2 abolished the asymmetry of Vam6 between mother and daughter cells (Figure 4J). Notably, while a strong asymmetry in vacuole acidification between daughter and mother cells was observed in both wild-type and *mob2ts* mutants at permissive temperatures, this difference was largely eliminated in *mob2ts* mutants under restrictive conditions without affecting vacuole inheritance (Figures 4K, 4L, and S4O). Together, our findings suggest that during asymmetric cell division, vacuoles and mitochondria migrate from the mother cell into the daughter cell, where Mob2 interacts with the vCLAMP component Vam6, post-translationally modifying it to stabilize it, thereby promoting Mito-Vac contacts and enhancing vacuole acidification in daughter cells (Figure 4C).

### Mitochondria-lysosome contacts facilitate lysosomal acidification in human cells

**Figure 5.**
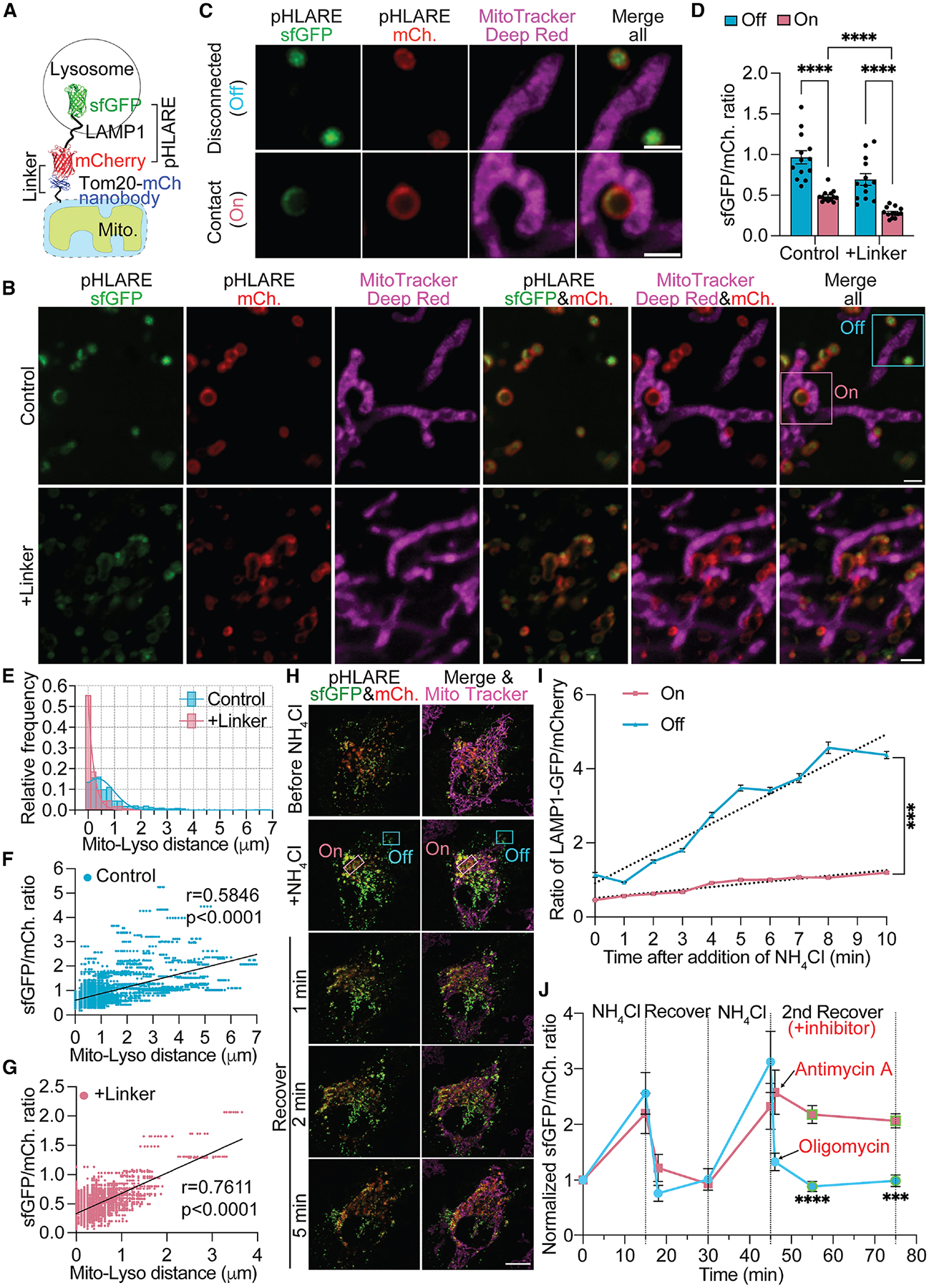
Mitochondria-lysosome contacts facilitate lysosomal acidification in HeLa cells (A) Schematics of the pHLARE and the engineered Mito-Lyso linker. (B and C) Representative super-resolution microscopy images of HeLa cells with and without Mito-Lyso linker (linker). Scale bar, 1 μm. (D) Comparison of lysosomal acidification in lysosomes located close to (<0.15 μm, “On”) and far from (>1 μm, “Off”) mitochondria in control and Mito-Lyso linker expressing (linker) HeLa cells. (E–G) Quantification of the distance between mitochondria and lysosome (E), as well as the Pearson correlation between Mito-Lyso distance and lysosomal acidification (F and G), in control and Mito-Lyso linker-expressing HeLa cells. (H) Representative images of lysosome pH neutralization and re-acidification in control HeLa cells. Scale bar, 10 μm. (I) Quantification of the acidification changes in lysosomes On or Off mitochondria during NH_4_Cl neutralization step. (J) Quantification of acidification changes in the entire lysosome pool within a cell during recovery, with and without inhibitors (control). Antimycin A or oligomycin was added during the second recovery phase.

Given the evolutionary conservation of both vacuole/lysosome acidification mechanisms (via V-ATPase) and the contacts between mitochondria and vacuole/lysosome,^28,29^ we then asked whether mitochondria-lysosome contacts play a similar role in the acidification of lysosomes in cultured human cells. To examine the difference in lysosome acidification, we used pHLARE, which was developed previously to report lysosomal acidification.^49^ pHLARE encodes rat lysosomal-associated membrane protein 1 (LAMP1), a lysosome transmembrane protein, tagged on the luminal side with an acid-sensitive GFP (sfGFP) and fused to mCh on the cytosolic side (Figure 5A). We confirmed that the ratio of sfGFP to mCh responded to acidification changes in the lysosome: lower pH resulted in a lower sfGFP/mCh ratio, while higher pH exhibited higher sfGFP/mCh ratio (Figure S5A). We then generated a stable HeLa cell line expressing pHLARE to examine the lysosomal acidification difference under various conditions and used MitoTracker Deep Red to monitor the mitochondria-lysosome (Mito-Lyso) contacts. Lysosomes are highly dynamic inside cells with most Mito-Lyso contacts lasting only 1–2 min.^28,29^ Our data showed a strong correlation between the distance from mitochondria to lysosomes and lysosome acidification: most lysosomes stably attached to mitochondria (“On”) exhibited a lower sfGFP/mCh ratio (more acidic), while those far away from the mitochondria (“Off”) usually showed higher sfGFP/mCh ratio (less acidic) (Figures 5B–5D and 5F; Video S1). A similar correlation was noted in MCF7 cells and primary human fibroblast IMR90 cells (Figures S5B and S5C).

The acidification status of some lysosomes did not correlate with their contact with mitochondria, likely due to the dynamic nature of mitochondria-lysosome contacts.^28,29^ Some lysosomes in contact with mitochondria may have already spent 1–2 min acidifying, while others may have just arrived at the mitochondrial surface to begin acidification. Similarly, lysosomes detached from mitochondria may have recently completed acidification, while others have been away from mitochondria for a significant time. To further interrogate this correlation, we introduced an engineered linker between mitochondria and lysosomes (Mito-Lyso linker) expressed from Tet-On promoter to enhance and stabilize the organelle contacts, using a plasmid expressing both Tom20tm-mCh nanobody and pHLARE (GFP-LAMP1-mCh) (Figure 5A). Echoing our observations in yeast, transient expression of this Mito-Lyso linker brought the two organelles closer together, reduced the mitochondria-lysosome distance, and slowed lysosome movement (Figures 5B–5E and S5D; Video S2). This increased interaction was accompanied by a stronger correlation between Mito-Lyso distance and lysosomal acidification, along with enhanced lysosomal acidification (Figures 5D, 5F, 5G, and S5E). A similar improvement in correlation was noted in MCF7 cells and primary human fibroblast IMR90 cells expressing Mito-Lyso linker (Figures S5F and S5G).

To complement the Mito-Lyso linker gain-of-function approach, we next investigated whether endogenous regulators of mitochondria-lysosome contacts influence lysosomal acidification. RAB7a, a small GTPase that promotes Mito-Lyso contact formation, and TBC1D15, a Rab7-GTPase activating protein (GAP) that antagonizes these contacts, were targeted using small interfering RNA (siRNA).^28,29^ RAB7a knockdown reduced mitochondria-lysosome contacts and impaired lysosomal acidification (Figures S5H–S5L). Conversely, TBC1D15 knockdown increased these contacts and enhanced lysosomal acidification, as indicated by a decreased sfGFP/mCh ratio (Figures S5H–S5L). These findings support a model in which mitochondrialysosome contacts promote lysosomal acidification and demonstrate that contact-site regulators can modulate lysosomal pH in mammalian cells, consistent with our observations in yeast.

To further investigate the role of mitochondria in lysosomal acidification, we conducted a lysosomal re-acidification assay by neutralizing lysosomes in HeLa cells with ammonium chloride (NH_4_Cl).^50^ To help track individual lysosomes during the experiment, we introduced a low concentration of microtubule inhibitor nocodazole to limit lysosome motility and mixing (Video S3). Upon addition of NH_4_Cl, most lysosomes were instantly neutralized, as indicated by an increased sfGFP to mCh ratio (Figure 5H). Interestingly, we observed that the lysosomes situated further away from mitochondria (Off) underwent a quicker and more significant loss of acidification compared with those on mitochondria (On) (Figures 5H and 5I). Following the NH_4_Cl washout, the lysosome swiftly re-acidified within 2–5 min. Once the cells fully recovered, we repeated NH_4_Cl neutralization and tested lysosomal re-acidification with inhibitors. Antimycin A, but not oligomycin, blocked the lysosome re-acidification after NH_4_Cl washout (Figure 5J). These results suggest that mitochondrial contact and ETC activity, as observed in yeast, contribute to lysosome acidification in human cells.

### Reduced mitochondria-lysosome contacts in human senescent cells contribute to lysosome defects and senescence-associated phenotypes

**Figure 6.**
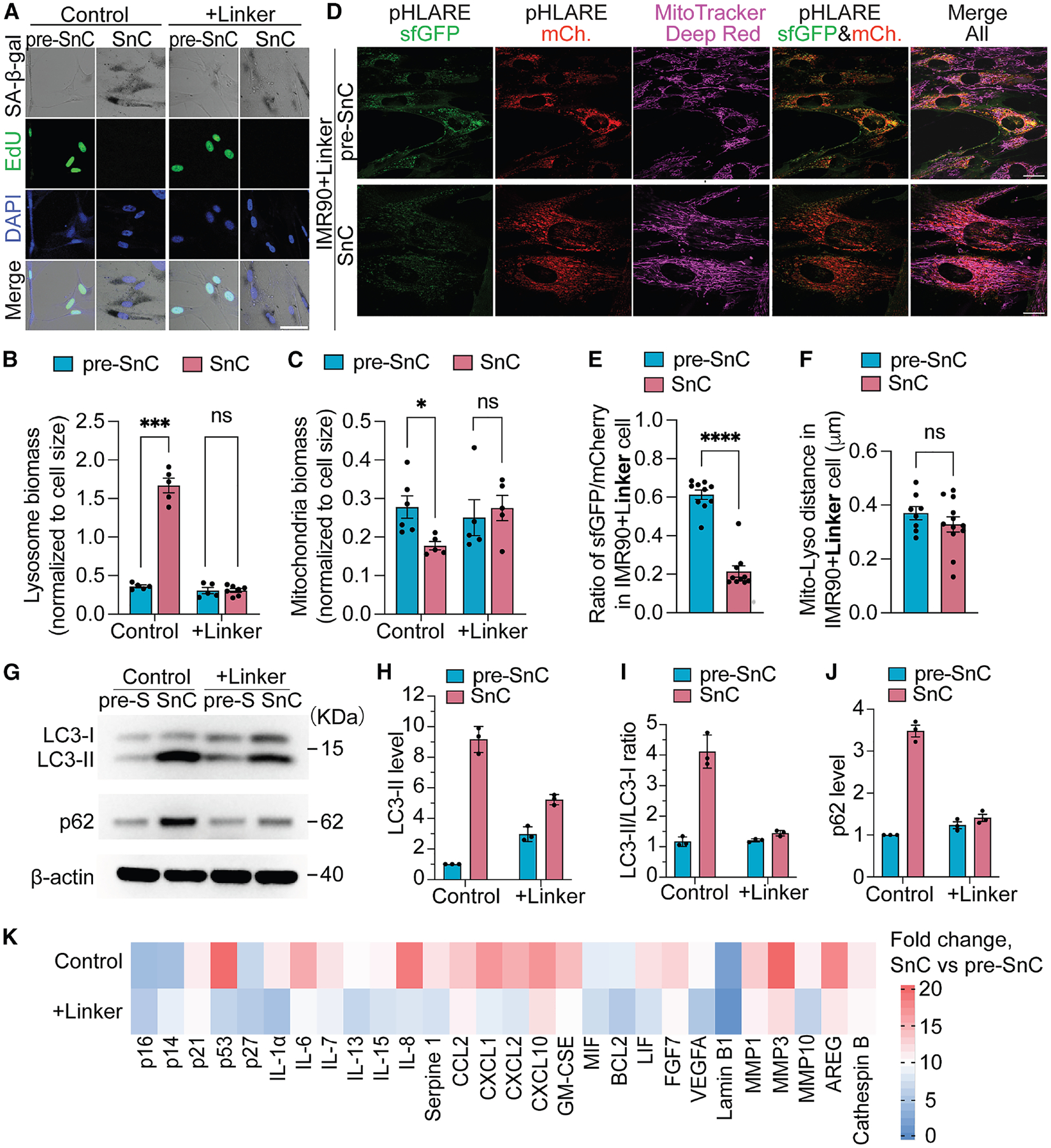
Reduced mitochondrial-lysosome contacts in human SnCs contribute to lysosome defects and SASP induction (A) Characterization of pre-SnC and SnC of IMR90 with/without Mito-Lyso linker. Pre-SnC, pre-senescent cell; SnCs, senescent cells. Scale bar, 100 μm. (B and C) Quantification of lysosomal (B) and mitochondrial (C) biomass in pre-senescent and senescent IMR90 cells with or without the Mito-Lyso linker, normalized to cell size. Control, wild-type IMR90 cells. +Linker, Mito-Lyso linker-expressing IMR90 cells. Same for other figures. (D–F) Representative images and quantifications showing lysosome acidification (D and E) and Mito-Lyso distance (F) in pre-SnC and SnC of IMR90 expressing the Mito-Lyso linker (Linker) as in Figure 5A. Scale bar, 20 μm. (G–J) Representative immunoblot and quantification of LC3-I, LC3-II, and p62 in pre-SnC and SnC of IMR90 with/without Mito-Lyso linker, normalized to actin loading control. (K) Heatmap of reverse-transcription quantitative PCR (RT-qPCR) results showing changes in SASP gene expression in IMR90 SnCs with and without the Mito-Lyso linker, normalized to their pre-senescent counterparts.

Similar to the aging-associated loss of vacuole acidification in yeast, lysosome de-acidification and dysfunction are well-established hallmarks of human senescent cells (SnCs).^51,52^ Given the conserved role of mitochondria in vacuole/lysosome acidification and the significant impact of Mito-Vac contacts on vacuolar acidification defects in aged yeast, we investigated whether Mito-Lyso contacts contribute to the loss of lysosomal acidification in human SnCs. We confirmed the loss of lysosomal acidification in senescent IMR90 cells induced by ionizing radiation—a classical model of SnC—using lysosome-specific, pH-sensitive ratiometric fluorescent probes (Figures 6A and S6A–S6C).^53^ In addition to impaired lysosomal acidification, these SnCs exhibited a marked increase in lysosome numbers, leading to a densely packed cytosol filled with lysosomes, consistent with a previous report^54^ (Figure 6B). By contrast, wild-type control SnCs displayed a reduced mitochondrial network and biomass (Figure 6C). This disproportionate expansion of lysosomes, without a corresponding increase in mitochondrial content, likely restricted mitochondria-lysosome interactions in wild-type control SnCs, thereby increasing the distance between these organelles and reducing their contact frequency (Figures 6C, S6B, and S6D).

Previous work has shown that Parkin positively regulates Mito-Lyso contacts and that its loss partially reduces these contacts.^55^ When we examined Parkin expression levels in wild-type IMR90 cells during cellular senescence, we found it to be significantly induced, likely reflecting a compensatory response to the reduced Mito-Lyso contact in SnCs (Figure S6E). However, this increase of Parkin expression levels in SnCs did not rescue the dramatically reduced Mito-Lyso contact compared with pre-SnCs (pre-SnC) (Figures S6B and S6D). Because Parkin has multiple functions beyond regulating Mito-Lyso contact,^56^ we turned to the engineered Mito-Lyso linker to test whether the reduced contact between mitochondria and lysosomes contributed to the loss of lysosomal acidification in SnCs. We introduced the Mito-Lyso linker into IMR90 cells and subjected them to the same dose of radiation as wild-type control cells. We confirmed that these Mito-Lyso linker-expressing IMR90 cells established cellular senescence, as shown by SA-β-galactosidase (β-gal) assay results and the lack of EdU incorporation, similar to control SnCs (Figure 6A). In contrast to the senescence-associated loss of lysosomal acidification in control SnCs, Mito-Lyso linker-expressing (+linker) SnCs showed a significant enhancement of lysosomal acidification compared with pre-SnCs expressing the same Mito-Lyso linker, as indicated by the reduction of sfGFP/mCh ratio (Figures 6D and 6E). Consistent with the effect of Mito-Lyso contact on lysosomal acidification, these Mito-Lyso linker-expressing SnCs maintained Mito-Lyso contacts/distances at levels comparable to their pre-SnCs (Figures 6D and 6F). This was likely due to the co-expansion of both lysosomal and mitochondrial populations, which scaled with the increase in cell size in these Mito-Lyso linker-expressing SnCs (Figures 6B and 6C). Notably, the density of lysosomes was significantly lower in these Mito-Lyso linker-expressing SnCs compared with control SnCs, which were densely packed with lysosomes in the cytosol (Figure 6D vs. Figures 6B and S6B). This suggests that the massive increase in lysosome biogenesis in control SnCs is likely a response to their lysosomal defect, as indicated by a previous report.^54^ Consequently, the ability of Mito-Lyso linker-expressing SnCs to maintain lysosomal acidification reduced the demand for increased lysosome biogenesis compared with control SnCs without a Mito-Lyso linker. These findings support a role for Mito-Lyso contacts in lysosomal acidification and suggest that preserving Mito-Lyso contacts during senescence is sufficient to prevent lysosomal acidification defects in SnCs.

We then investigated whether preventing lysosomal acidification defects in SnCs has any biological consequences. It is known that lysosomal acidification defects in SnCs reduce autophagic flux.^57,58^ Consistently, control SnCs showed increased level of the autophagy receptor p62, a substrate for autophagic turnover, and exhibited a higher LC3-II/LC3-I ratio compared with their pre-senescent controls, mirroring the autophagic blockage effect observed when pre-SnCs are treated with bafilomycin A (BafA) (Figures 6G–6J and S6F–S6I). In contrast, Mito-Lyso linker-expressing SnCs, which maintained functional lysosomal acidification, exhibited much less accumulation of LC3-II and p62 compared with control SnCs, suggesting that preserving lysosomal acidification enhances lysosomal function and autophagic flux in SnCs (Figures 6G–6J). Indeed, the benefit of the Mito-Lyso linker was erased by treating these cells with BafA, which inhibits lysosome acidification (Figures S6F–S6I). Beyond changes in autophagic flux, SnCs are also known to undergo structural and functional alterations in other organelles, including increased nucleolar number/size and altered Golgi apparatus structures.^51,59–61^ While expression of the Mito-Lyso linker had limited effects on nuclear and nucleolar number/size in SnCs (Figures S6J–S6M), it partially rescued the structural and size changes of the Golgi apparatus in these cells (Figures S6N and S6O).

The rescue of Golgi apparatus and its role in secretion prompted us to examine another well-established phenotype of SnCs: the senescence-associated secretory phenotype (SASP). The induction of SASP factors is a key mechanism driving the deleterious effects of SnCs, including inflammaging and tissue damage.^51,62–64^ We confirmed by reverse-transcription quantitative PCR (RT-qPCR) that many well-established SASP factors are highly induced in control SnCs, including inflammatory cytokines interleukin (IL)-1α, IL-6, IL-8, and CXCLs (Figure 6K). Surprisingly, the majority of these SASP factors were not induced in Mito-Lyso linker-expressing SnCs, including IL-1α, IL-6, IL-8, and CXCL1/2 (Figure 6K). Although several other SASP factors, such as CCL2, CXCL10, and MMP1/3/10, were still induced in Mito-Lyso linker-expressing SnCs, their induction levels were significantly lower compared with control SnCs (Figure 6K).

**Figure 7.**
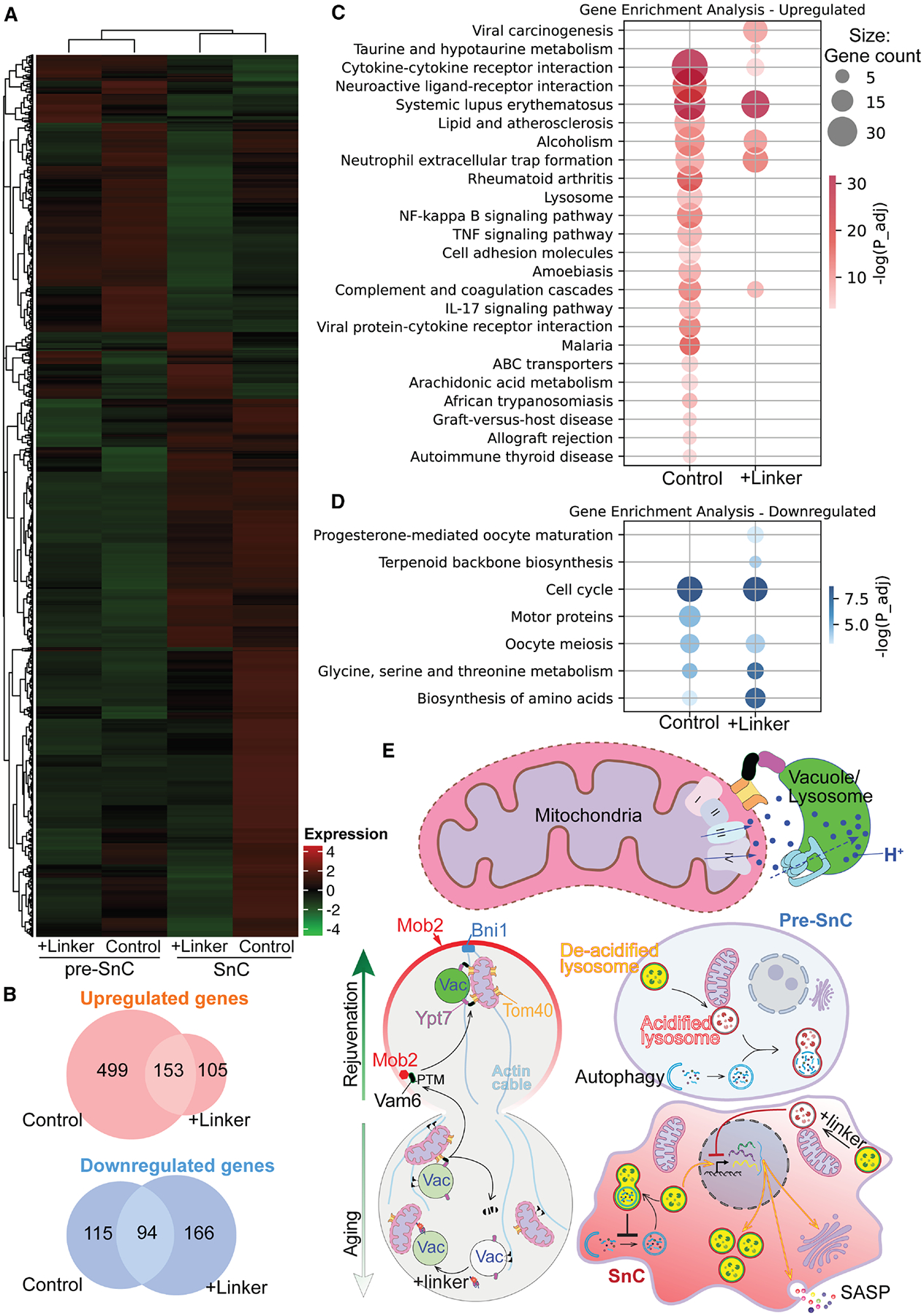
Preserving mitochondria-lysosome contacts in SnCs blocks multiple characteristic pathways of cellular senescence (A) RNA-seq showing overall gene expression changes between pre-senescent and senescent IMR90 cells with/without Mito-Lyso linker expression. (B) Venn diagram of genes upregulated and downregulated in senescent IMR90 cells with/without Mito-Lyso linker expression. (C and D) Kyoto Encyclopedia of Genes and Genomes (KEGG) pathways identified by gene set enrichment analysis (GSEA) of the upregulated (C) and downregulated (D) gene groups in senescent IMR90 cells with/without Mito-Lyso linker expression. (E) Mitochondrial MMP and their membrane contacts with vacuole/lysosome play a crucial role in the acidification of vacuoles and lysosomes by facilitating the flow of proton from mitochondria to vacuole/lysosome (top). This mitochondria-dependent mechanism of vacuole/lysosome acidification has important roles in the replicative aging and rejuvenation of yeast (bottom left) and cellular senescence of human cells (bottom right).

These substantial changes in autophagic flux and SASP prompted further investigation into additional pathways altered by the Mito-Lyso linker and lysosomal acidification in SnCs. Transcriptome analysis revealed significant differences between Mito-Lyso linker-expressing SnCs and control SnCs (Figure 7A), with the control SnCs exhibiting more than twice as many upregulated genes (Figure 7B). Although reduced cell-cycle activity and diminished amino acid metabolism are shared features, control SnCs exhibited classic signatures of senescence, including the induction of inflammatory cytokines, activation of the nuclear factor κB (NF-κB) signaling pathway, and upregulation of lysosome-related genes, whereas Mito-Lyso linker-expressing SnCs showed much less inflammatory gene induction and no activation of NF-κB signaling or lysosome-related genes (Figures 7C and 7D). The SenMayo senescence gene set, comprising nine distinct protein classes across 125 genes, was used to identify SnCs across various tissues and species with high accuracy.^65^ Comparison of the SenMayo network between control and Mito-Lyso linker-expressing SnCs confirmed significant differences across several classes, including most cytokines, many growth factors (e.g., IGF1, PGF, VEGFC, and FGF1), and many protease inhibitors (Figure S7). These findings suggest that preserving Mito-Lyso contact prevents lysosomal acidification defects during senescence and fosters a “quiet” senescent state.

## DISCUSSION

Together, our data support a model in which mitochondria contribute directly to vacuole/lysosome acidification by supplying ETC-derived protons through mitochondria-vacuole/lysosome contact sites (Figure 7E). Consistent with our findings, while our study was in the final stages of revision, an independent study reported a similar mechanism: protons pumped out by the ETC contribute to lysosome acidification through membrane contacts.^66^ In both aged yeast cells and senescent human cells, reduced organelle contact contributed to the loss of vacuolar/lysosomal acidification, whereas restoring contact with an engineered linker reversed this defect. Moreover, the asymmetric cell division and natural rejuvenation process in the daughter yeast cells increase the contacts between these two organelles, thereby re-acidifying the previously alkalized vacuoles inherited from aged mother cells.

These findings revise the traditional view that lysosomes and vacuoles are acidified primarily by capturing freely diffusible cytosolic protons. That model does not readily explain the spatial heterogeneity of lysosome/vacuole pH observed in yeast and mammalian cells. Our results instead suggest that mitochondrial proton output is an important local proton source, while V-ATPase remains the machinery that imports those protons into the lumen. Consistent with this interpretation, V-ATPase abundance did not decline during yeast replicative aging, and old vacuoles could be rapidly re-acidified when mitochondriavacuole contact was restored, indicating that early-life de-acidification is not simply caused by irreversible loss of V-ATPase function. Although an age-associated reduction of V-ATPase mRNA expression was reported in *C. elegans*,^67^ quantitative proteomics have shown that various V-ATPase subunits are not significantly changed at the protein levels during *C. elegans* aging.^68^

Our data further suggest that membrane contact sites enable proton transfer by placing V-ATPase near the mitochondrial outer membrane in a crowded, highly buffered cytosol where free protons are likely to be rapidly captured by competing molecules. This framework also offers an explanation for lysosomal pH heterogeneity: lysosomes in contact with mitochondria acidify faster and more strongly than those farther away. Since mitochondria-lysosome contacts are dynamic, contact frequency and duration may tune lysosomal pH across the cell. In addition to lysosomes, some endosomes are also acidified, though their luminal pH is less acidic than that of lysosomes. Interestingly, several studies have suggested contacts between mitochondria and endosomes, as well as secretory granules.^69–71^ It is plausible that different organelles employ distinct combinations of proton sources—both mitochondrial and cytosolic. Organelles in direct contact with mitochondria may achieve greater acidification by drawing protons from mitochondria, whereas vesicles relying solely on cytosolic protons may be limited by the low availability of free H^+^ in the cytosol.

Finally, the senescence results indicate that senescence-associated signaling likely impairs mitochondria-lysosome contact despite compensatory lysosome expansion, whereas an engineered Mito-Lyso linker is insulated from such changes and therefore preserves lysosomal acidity. Preserving lysosomal acidification improved autophagic flux and markedly reduced major inflammatory SASP factors, supporting a causal link between lysosomal dysfunction and the inflammatory state of SnCs. More broadly, our study identifies a conserved mitochondria-to-lysosome/vacuole axis that helps explain age-associated de-acidification from yeast to human cells and suggests that restoring organelle coupling or lysosomal acidification may be a useful strategy for conditions marked by lysosomal dysfunction.

### Limitations of the study

The precise mechanisms by which senescence disrupts Mito-Lyso contacts in human cells remain unclear. Furthermore, while our findings reveal a Bni1- and Mob2-dependent mechanism for vacuole re-acidification during asymmetric cell division, additional research is needed to fully elucidate the remaining mechanistic details of this vacuole rejuvenation process. Finally, it is important to note that the engineered Mito-Lyso tether lacks the dynamic regulation of endogenous contact sites, that ETC inhibitors may have side effects, and that the imaging modalities used in this study provide only relative distance and proximity between organelles and do not resolve the precise physical distance between their membranes. It is also possible that different tissues exhibit varying degrees of mitochondria-lysosome contact and reliance on mitochondria for lysosomal acidification. Future studies will be necessary to address these questions.

## STAR★METHODS

### RESOURCE AVAILABILITY

#### Lead contact

The lead contact is Chuankai Zhou (kzhou@buckinstitute.org).

#### Materials availability

All unique/stable reagents generated in this study are available from the lead contact with a completed materials transfer agreement.

#### Data and code availability

- The published article includes all datasets generated or analyzed during this study. RNA sequencing data have been deposited in NCBI’s Gene Expression Omnibus (GEO) under accession number GEO: GSE292573. Original raw data are deposited in Mendeley Data: https://doi.org/10.17632/j99xc458mz.1 and are publicly available as of the date of publication.
- This paper does not report original code.
- Any additional information required to reanalyze the data reported in this paper is available from the lead contact upon request.

### EXPERIMENTAL MODEL AND STUDY PARTICIPANT DETAILS

#### Yeast strains and culture condition

Yeast strains used in this study are based on the BY4741 strain background. Genetic modifications were performed with PCR mediated homologous recombination^72^ and genotyped with PCR to confirm correct modification and lack of aneuploid for the chromo-some that gene of interest is located. The GFP-tagged strains were from the yeast GFP collection.^73^ The mutant strains were generated by PCR amplify KANMX cassette from existing mutant strains (Yeast Knockout Collection, GE Dharmacon) or pFA6a-KanMX plasmid to transform BY4741 or the strains based on BY4741. Expression of proteins from integration plasmids was done by integrating the linearized plasmid into TRP1 locus. For some strains, an empty integration plasmid was first integrated into the TRP1 locus to provide the required sequences, such as the AMP gene in the backbone of plasmid, for further integration of other plasmids. The strains, plasmids, primers, antibodies, and chemical reagents used in this study are listed in key resources table.

For confocal imaging, cells were grown at 30°C in SC glucose medium (790 mg/L of complete supplement mixture (CSM) from Bioworld, 6.7 g/L yeast nitrogen base, 2% glucose) or YPD (10 g/L yeast extract from BD, 20 g/L peptone from BD, and 20 g/L glucose) overnight to OD_600_ ~ 1, and refreshed for additional 2–3 hrs before imaging. For the strains with Gal promoter-driven proteins, cells were grown at 30°C in SC or YEP with 2% raffinose medium overnight to OD_600_~0.5 and induced by adding 2% galactose (gal-induction). The gal-induction time depends on the purpose of experiments. Usually pGal promoter-driven protein expression reaches a plateau after switch from 2% raffinose medium to 2% galactose medium for >3 hrs at 30°C, for example, in Figures 2 and S2, the gal-induction time for Vam6 and (or) Ypt7 is 3 hr, gal-VPS10-mCh nanobody is 30–40 minutes. If switched from YPD medium directly to YP-galactose, it takes longer time to reach the plateau, as the cells have to deplete glucose competitive effect first before taking in galactose, for example, in Figure 3B, the gal induction time for gal-Tom70-GFP_11_ is 3 hr. Gal-induction was terminated by adding 2% glucose for 30 minutes before other treatment or imaging. All media were prepared by autoclaving the solution without glucose/raffinose/galactose for 20 minutes at 121°C and add filtered carbon source as indicated.

#### Mammalian cell lines and culture condition

Human cells were maintained in Dulbecco’s Modified Eagle’s Medium with L-Glutamine and 4.5 g/L Glucose (Corning 10013CV), supplemented with 10% FBS (Gibco) and 1% penicillin-streptomycin (Gibco) in 60×15 mm tissue culture treated dishes at 37 °C in an atmosphere of 5% CO_2_. Transient transfections were performed with PolyFect Transfection Reagent (QIAGEN 301105) for HeLa and MCF7 cells, Lipofectamine^™^ LTX Reagent (Invitrogen 15338030) for IMR90 cells according to the manufacturer’s instruction. Purified plasmids used for transfection are listed in key resources table. Stable cell lines were generated by selection with 400 μg/mL G418 for two weeks after transfection. Single-cell clones were isolated by limiting dilution and validated by fluorescence microscopy. MCF7 cells stably expressing pHLARE plasmid were provided by Dr. Diane L Barber. IMR90 cells were originally obtained from Judith Campisi lab. Pre-senescent IMR90 cells are with less than 15–17 passages. Senescent IMR90 cells are induced by exposing to a single dose of 10 Gray ionizing radiation (IR) for 9 minutes according to the treatment condition in Campisi lab. After IR, placed cells back to the incubator and changed medium every 48 hrs. Assays for senescent cells were done 10 days after IR.

Transfection of cells with siRNA was performed using RNAiMAX from Invitrogen following the manufacturer’s instructions. Rab7a siRNA was purchased from IDT: sense sequence 5^′^-GGAUGACCUCUAGGAAGAATT-3^′^ and antisense sequence 5^′^-UUCUUCCUA GAGGUCAUCCTT-3^′^.

TBC1D15 siRNA was from ThermoFisher (AM16708). Control RNA was used as a negative control: sense sequence 5^′^-ACUUC GAGCGUGCAUGGCUTT-3^′^ and antisense sequence 5^′^-AGCCAUGCACGCUCGAAGUTT-3^′^.

For microscopy purpose, cells were seeded in 35 mm Confocal imaging dishes (VWR 75856–742) for 24 hr, then moved to a microscope chamber at 37 °C with 5% CO_2_ for drug treatment and imaging. To continually track the same live cells for a period during imaging, the cell positions in the dishes were marked with the microscope stage control before changing medium or buffer carefully. Mitochondria were stained with MitoTracker Deep Red FM (Invitrogen M22426) by adding 1 μM dye into the dish (2 mL medium) and incubate for 10 minutes. To reduce the organelle movement and track single lysosome during timelapse movies, 0.5 μg/ml Nocodazole (Sigma M1404 dissolved in DMSO) was added 15 minutes before imaging.

### METHOD DETAILS

#### Microscopy

Confocal images were acquired using a Carl Zeiss LSM-510 Confocor 3 system with 100× 1.45 NA Plan-Apochromat objective and a pinhole of one airy unit. The system was driven by Carl Zeiss AIM software for the LSM 510 meta. 488/561 nm laser was used to excite GFP/RFPs, and emission was collected through the appropriate filters onto the single photon avalanche photodiodes on the Confocor 3. GFP images were acquired through a 505–540 nm filter, and RFP images were acquired with a 580–610 nm filter on the Confocor 3. All images were acquired in a multi-track, alternating excitation configuration so as to avoid bleed-through. All image processing was performed in the Image J software (NIH, Bethesda, MD). For visualization purposes, images were scaled with bilinear interpolation was used for figures. The imaging experiments were done with >3 biological samples for each strain/treatment in each repeat performed on the same day. We quantify the cells from these repeats and then verify the results again by an independent repeat done on a different day. To get time-lapse movies, yeast cells are placed on a thin agarose gel pad between the slide and coverslip and fill the gap with medium and seal with Valap (1:1:1 mix of vaseline, lanolin and paraffin) to support cell growth and keep the image focus.

Super resolution microscopy was performed on live yeast and mammalian cells using Nikon Ti2 SoRa system (120nm x-y resolution). However, the high laser intensity required for super-resolution imaging resulted in significant photobleaching and phototoxicity, particularly to mitochondria in live cells. In mammalian cells, mitochondria became fragmented after 2–5 seconds of exposure during super-res data acquisition, making it unsuitable for long-term time-lapse imaging. To minimize photodamage, long-term tracking of the same cells was instead conducted using a Zeiss confocal microscope equipped with an avalanche photodiode (APD) detector, which is a single-photon-sensitive detector (Figure 5H). The APD offered high sensitivity at low laser power, allowing for time-lapse imaging while minimizing phototoxicity and bleaching effects. For Figure 4A, to mitigate photodamage caused by continuous laser exposure, super-resolution imaging was conducted at discrete time intervals rather than in a continuous time-lapse sequence to monitor cell budding following release from synchronization with α-factor.

#### Drug treatment and key reagents

Quinacrine dihydrochloride (Sigma Q3251) dissolved in H_2_O to 20 mM as stock solution. Tetramethylrhodamine, Methyl Ester, Perchlorate (TMRM) (Thermo Fisher Scientific T668) dissolved in DMSO to 1 mM as stock solution, 1:10,000 for 15 minutes for mitochondrial membrane potential detection and quantification. Calcofluor White Stain (Sigma-Aldrich 18909–100 ML-F) was diluted 200 folds in PBS and incubated with cells for 5 minutes at room temperature, followed by three washes with PBS. β-estradiol (Sigma E8875) dissolved in ethanol to 10 μM as stock is used at 100 nM as final concentration to induce Tom70 overexpression driven by Z3 promoter during old cell purification. Antimycin A (Sigma A8674) dissolved in DMSO to 10 mM as stock is used at 10 μM to inhibit ETC. Oligomycin (Sigma 75351) dissolved in ethanol to 10 mM is used at 10 μM to inhibit ATPase. For Figure 1F, wild-type cells were cultured in YPD and treated with antimycin A or oligomycin for 120 minutes before quinacrine staining. For Figure 5J, mammalian cells were tracked for 30 minutes after adding antimycin A or oligomycin during recovery. α-Factor (Zymo Research Y1001) 10 μg/ml was added for 3 hr to synchronize yeast cells at 21°C due to longer doubling time at this temperature. MitoTracker Deep Red FM (Invitrogen M22426) was added to mammalian cell culture at a final concentration 0.5 nM and incubated at 37°C for 15 minutes to stain mitochondria. Nocodazole (Sigma M1404) dissolved in DMSO to 1 mg/ml is used at 0.5 μg/ml for 15 minutes to inhibit microtubule assembly thereby reducing organelle movement in mammalian cells. A final concentration of 50 mM NH_4_Cl was added to neutralize lysosomes. GFP antibody is from Sigma (SAB4301138). Flag antibody is from Sigma (F3165). LC3 antibody is from Cell Signaling Technology (mAb #12741). p62 antibody is from Abcam (ab109012). The nucleolus was stained with the BioTracker^™^ rRNA Live Cell Probe (Sigma, SCT089). The Golgi apparatus was visualized by immunofluorescence staining using an Alexa Fluor^®^ 647-conjugated GM130 antibody from Abcam (ab195303).

#### Quinacrine staining of vacuole pH

Quinacrine staining was performed as previously described (Hughes and Gottschling 2012).^16^ 2 × 10^6^ mid-log phase young cells or purified old cells were washed once in YPD supplemented with 100 mM HEPES, pH 7.6. These cells were then resuspended in 100 μl of YPD/HEPPES containing 200 μM quinacrine (Sigma Q3251). Cells were incubated at 30 °C for 10 minutes followed by 5 minutes on ice. Cells were then quickly (within 2 minutes) washed twice with ice-cold HEPES buffer (100 mM, pH 7.6) containing 2% glucose before being resuspended in the same buffer for imaging. Cells that have been stained were kept on ice for no more than 30 minutes before imaging. To avoid quinacrine staining variation, cells from different samples were mixed in a 1:1 ratio (OD_600_) and stained together (different samples have different markers). For Figure 2K, the samples of *in vitro* assay were incubated with 100 μM quinacrine on ice for 15 minutes without washing before imaging.

#### Isolation of Cells with Advanced Replicative Age

Yeast cells were collected from fresh overnight culture and washed twice with cold PBS, pH 8.0. About 4 ×10^7^ mid-log cells were briefly concentrated by 3,000 g for 20 sec, followed by three washes of cold sterile PBS pH 8.0. Cells were labeled with 2 mg/ml EZ-Link^™^ Sulfo-NHS-LC-Biotin (Thermo, 21335) at room temperature in the same PBS for 30 minutes with gentle shaking. These cells were used as M-cells. The M-cells were then washed three times with cold PBS, pH 7.2, to get rid of free biotin. These M-cells were resuspended in 500 ul PBS pH7.2 and mixed with 30 μl BioMag magnetic streptavidin beads (Polysciences, 84660) for 60 minutes at room temperature and loaded into 6-well cell culture plate (Genesee Scientific, 25–100) and culture cell in the shaker (Biomega, Incu-Shaker Mini) at 30°C with minimum speed 100 rpm to maintain cells resuspended. Change fresh medium every 4 hr. To change medium, placing curved grade N52 magnets at the bottom of the 6-well plate to attract the magnetic streptavidin beads-bound M-cells, then remove the young cells along with the old medium and replace with fresh medium. After changing medium, remove the magnets and put the 6-well plate back to the shaker. After culture for 36–48 hrs, collect the M-cells with magnets and stain bud scars with calcofluor white (Sigma 18909, add 7 μl to 1 ml cell suspension). If need fixed cells, add 1% formaldehyde for 30 minutes at room temperature to fix cells and washed three times with PBS, pH 7.4, then stain fixed cells with calcofluor white. The stained cells were imaged with confocal to collect images of the bud scars and the other channels of interests. Cells with more than 12 bud scars were treated as old cells.

To induce Mito-Vac linker in old cells: cells were initially cultured in YPD medium until they reached advanced ages. Before harvesting old cells, switch to YP-Raffinose medium for 3 hr to deplete glucose, then add 2% galactose into medium to induce linker expression for 1 hr, followed by adding 2% glucose for 30 minutes to stop induction.

To induce split-GFP in old cells: cells were initially cultured in YPD medium until they reached advanced ages. Before harvesting old cells, switch to YP-Galactose medium directly for 3 hrs to induce Tom70-GFP_11_ expression.

#### FM4–64 staining

Vacuole were stained with FM4–64 as previously described.^74^ Briefly, FM4–64FX 100 mg (Thermo Fisher Scientific F34653) was dissolved in 32 μl DMSO (4 mM) as stock solution, and 1:1,000 dilution was used to stain 1 ml cells in their growing medium for 1 hr in dark. Cells were then diluted into 10 ml fresh medium and incubated for 5 hrs before imaging.

#### 3D in silico simulation of proton diffusion and capture

To model proton diffusion from the mitochondrial surface to the vacuole in a crowded cytosolic environment, we implemented a three-dimensional Brownian motion simulation in Python. The simulation volume was a 2 μm cube, with the mitochondrial outer membrane modeled as a flat proton-emitting surface at the base (z = 0). A spherical vacuole of radius 0.5 μm was positioned at varying membrane-to-membrane distances between 1 and 500 nm. For each distance, 1,000,000 protons were released from random x–y positions on the mitochondrial surface. Protons underwent unbiased Brownian motion with a diffusion coefficient of 930 μm^2^/s, consistent with estimates of proton mobility in buffered cytoplasm.^75^ Motion was updated every 0.1 μs, and each proton trajectory was simulated up to 1 ms or until it encountered a capture, block, or escape event.

The vacuolar surface was populated with 6000 V-ATPase complexes, reflecting an estimated density of ~1900 molecules/μm^2^ in yeast vacuolar membranes.^6^ Each V-ATPase was modeled as a spherical capture site with a 5 nm radius. This value approximates the combined structural and electrostatic range of the proton uptake region in the V0 sector, based on cryo-EM studies of V-ATPase architecture and prior biophysical modeling of membrane-bound proton pumps.^76,77^ Cytosolic crowding was represented by ~12.5 million non-overlapping spherical (protein) crowders (radius 3.5 nm), occupying ~30% of the cytosolic volume, which was calculated by subtracting the vacuolar volume from the total simulation cube.^78^ Additionally, 60,000 phosphate ions (radius 1 nm) were randomly distributed throughout the same available cytosolic space. This number corresponds to a physiological concentration of ~50 mM phosphate, corrected for the cytosolic volume and excluding the vacuole.^7,79^ Crowder and phosphate positions were held constant across all simulations, ensuring that only proton release coordinates and vacuolar distance varied between runs. Simulations were executed serially due to instability with multiprocessing. Proton capture rates were highly reproducible across runs for each distance, confirming convergence and robustness of the simulation. Due to the small size of vacuole/lysosome, a few thousand protons are sufficient to fully re-acidify a neutralized lumen.

#### in vitro vacuolar acidification assay

The strains used for in vitro assay includes (**a**) VPS10-mCherry nanobody; (**b**) mCherry-Fis1tm; and (**c**) WT. Culture cells in YP-raffinose medium overnight. Refresh cells with fresh medium for 3 hrs, then add galactose for 2 hrs (to all three strains including WT) to induce nanobody expression, followed by adding glucose for 30 minutes to stop the induction. Harvest Cells by centrifuge at 3,000 g for 3 minutes. Wash with 100 mM Tris-H_2_SO4 (pH 9.4), then re-suspend cells in Tris-H_2_SO_4_ and add DTT to 10 mM (10 uL of 1M DTT into 1 mL). Rotate for 5 minutes. Wash twice with 1.2 M sorbitol digestion buffer, then re-suspend in Lallzyme MMX (Lodi Wine Labs) dissolved in digestion buffer (7 mg/mL). Then rotate at room temperature for 20 minutes. Pellet cells for 2 minutes at 3,000 g. Wash twice with chilled digestion buffer (1.2 M sorbitol, pH 7.5), carefully leave some soup so the cell pellet does not get washed away. Suspend the cells with prechilled lysis buffer (20 mM HEPES-KOH, PH 7.5, 0.6 M sorbitol, 150 mM KCl, 0.5 mM MgCl_2_) with protease inhibitors. Pipette up and down for 10 times on ice and incubate on ice for 5 minutes. Filter the cell lysate with 5 μm filter to open the cells without damaging the mitochondria (diameter of ~0.5 μm). The 0.6 M sorbitol is commonly used to prevent the lysis of mitochondria^80^ but inevitably causes the swelling of some mitochondria (round circles). Mix the lysates (**a**+**b**, and **b**+**c**) with a 1:1 ratio, and add ATP generating system (0.5 mM ATP (Sigma A1852), 20 mM creatine phosphate (Sigma 10621714001), and 35 U/ml creatine kinase (Sigma 10127566001)) incubate on ice for 10 minutes. Centrifuge at 12,000 g for 15 minutes at 4°C to promote the interaction and binding between the mitochondria and vacuole. This centrifugation alone did not cause mitochondria-vacuole association as shown by the control group with organelles from strain **b** and **c** (-linker in the Figure 2K). Resuspend pellets in 100 μl soup and add 2 mM NADH (Sigma 10128023001) and incubate on ice for 10 minutes, then add quinacrine (100 μM, half of the concentration used in *in vivo* assay to reduce background) to stain vacuole. Inhibitor antimycin A or oligomycin was added during the incubation with quinacrine.

#### Quantification of organelle distance in yeast

Inter-organelle distances (Mito-Vac and ER-Vac distance) were determined using a custom ImageJ macro as follows. Firstly, the vacuole was segmented by Gaussian blurring with a standard deviation of 1 pixel, rolling ball background subtraction with a radius of 10 pixels and then thresholding at the average intensity multiplied by 9. Next the other organelles were found by 1-pixel standard deviation Gaussian blurring, rolling ball background subtraction with a radius of 5 pixels, maximum intensity z projection and tracking with a “track max not mask” methodology. This methodology consists of repeatedly finding the maximum intensity in the image and masking the region around it before finding the maximum again until no pixels are above the chosen threshold as we did previously.^81^ For the organelles, the region diameter was 15 pixels with a threshold set at 10 times the average image intensity. These organelles were then tracked in the z dimension by finding the maximum z position within a column spanning 4.5 pixels from the tracked xy position. Finally, the minimum distances between the 3D organelle positions and the segmented vacuolar regions were found by brute force search from each organelle position until the nearest vacuolar voxel was found.

#### Immunofluorescence

Culture yeast cells in YPD medium overnight at 30 °C to either mid-log density (OD_600_~0.5) or high OD (OD_600_>2) as indicated in the figure. Refresh the cells for 3–4 hrs. Harvest cells by centrifuge at 3,000 g for 2 minutes. Wash once with 100 mM Tris pH 7.5. Re-suspend in 100 mM Tris pH 7.5 with 10 mM DTT and rotate for 2–3 minutes at room temperature. Wash twice with digestion buffer (0.1 M K_2_HPO_4_/KH_2_PO_4_ pH 7.5, 1.2 M sorbitol). Digest cell wall with 7 mg/ml Lallzyme MMX (Lodi Wine Labs) dissolved in digestion buffer, rotate for 5 minutes at room temperature. Wash three times with digestion buffer. Fix cells with 4% PFA digestion buffer for 30 minutes at room temperature on the bench. Wash 5 times with washing buffer (PBS, 1 mg/ml BSA, 0.3–0.5% TritonX-100). Add 2 μl Pma1 antibody (Thermo Fisher MA1–91567) to 1 mL washing buffer and incubate at 4°C overnight. Wash 5 times with washing buffer and add 1ul Goat anti-Mouse IgG (H+L) Secondary Antibody conjugated with Alexa Fluor 568 (Thermo Scientific A-11004) and incubate for 6–8 hrs at 4°C in dark. Wash 5 times with washing buffer before imaging the cells.

For GM130 Immunofluorescence, mammalian cells were fixed cells with 100% methanol for 5 minutes, permeabilized in 0.1% Triton X-100 for 5 minutes and then blocked in 1% BSA, 22.52 mg/mL glycine in PBST (PBS with 0.1% Tween 20) for 1 hr. The cells were then incubated with ab195303 at 5 μg/ml overnight at 4°C. Nuclear DNA was labelled in blue with DAPI.

#### Replicative lifespan (RLS) of yeast

Yeast replicative lifespans were measured in the Hao lab using microfluidics coupled with time-lapse microscopy as previously described.^82–85^ For Figure 3I, cells constantly expressing the low level of Mito-Vac linker (pZrc1-Zrc1-mCh nanobody and mCherry-Fis1tm) and control strain (mCherry-Fis1tm only) were loaded into cell trapping chambers in a microfluidic device with SC-glucose medium. The flow of SC glucose medium in the device was maintained by gravity throughout the entire experiment. Time-lapse phase images of aging mother cells were acquired by a Nikon Ti-E inverted microscope every 15 minutes for a total of 80 hours. The number of cell divisions of each mother cell until its death was manually counted from the time-lapse images.

#### α-Factor synchronization of temperature sensitive yeast cells bni1ts

Cells were cultured in YPD medium at 21°C overnight to OD_600_ 1. Refresh cells with fresh medium for 3 hrs at 21°C. Dilute cell culture to OD_600_ 0.2 and add α-factor (10 μg/ml) and incubate for 3 hrs to synchronize cells. Then pellet and wash cells with fresh medium for five times to remove α-factor. Resuspend cells in fresh medium and put back to 21°C for 15 minutes to release cells into cell cycle. Cells were then shifted to 30°C for 30 minutes followed by 30 minutes at 35°C to inactivate *bni1ts*. This stepwise inactivation was used to avoid heat shock response associated with 35°C when shift from 21°C.

#### Quantification of Mito-Vac overlapping in mother and daughter cells

3D images of organelles were acquired with confocal. The quantification was carried out on a single-cell basis by first defining the cellular boundary using YeastSpotter, a convolutional neural network-based algorithm for the segmentation of yeast microscopy images into single cells.^86^ After that, images were first blurred with a one standard deviation Gaussian filter. The cytoplasmic background was then subtracted with a rolling ball background subtraction with a radius of 10 pixels. For organelles, the image was then segmented with a threshold between 12 and 6 times the average intensity of each subtracted image. Segmented objects were then filtered by Analyze Particle to remove any objects with less than 5 pixels and converted into masks. These masks of organelles were then used to quantify the amount of overlapping between the organelles. The total area of vacuole mask (Vi) across the Z stacks within a cell and the mitochondrial mask falling/overlapping within the vacuole mask (MVi) across the Z stacks of that cell was measured for both mother and daughter cell in a dividing cell. The mitochondria overlapped with vacuole was normalized by vacuole size in the corresponding cell:

$$F=\frac{\sum_{i=1}^{n}MVi}{\sum_{i=1}^{n}Vi}$$

Where *i* is the individual focal plane of the Z stack images. For each dividing cell, this normalized Mito-Vac overlap (F) in the daughter was then compared with its mother cell to get the Bud/Mother ratio showed in Figure S4B.

#### SplitGFP assay

Cells with pGal-Tom70-GFP_11_ and Zrc1-GFP1–10 were culture in YPD medium in 6-well plate at 30 °C for 36 hrs to reach advanced ages. Before harvesting old cells, switch to YP-Galactose medium directly for 3 hrs to induce Tom70-GFP_11_ expression. This is because the direct Gal-induction on cells grown in glucose medium takes several hours to remove the glucose suppression and this 3 hrs-induction with galactose is not overexpression. GFP1–10 is tagged to the C-terminus of endogenous Zrc1, which is also a low abundant of vacuolar protein. FM4–64 was added when switch medium to stain vacuoles. Gal-induction was terminated by adding 2% glucose for 30 minutes. Harvest cells and stain bud scars with calcofluor white. All the strains including the controls (Zrc1-GFP and pGal-Tom70-GFP) were cultured in the same condition. The stained cells were imaged with confocal to collect images of the bud scars and the other channels of interests.

#### Quantification of Mito-Lyso distance and lysosome pH in mammalian cells

Mammalian cells with sfGFP-LAMP1-mCherry were cultured in 35 mm Confocal dishes (VWR, 75856–742) for 24 hrs. Stain mitochondria with MitoTracker Deep Red (1 μM) for 10 minutes before imaging. Place the dish to a microscope chamber at 37 °C with 5% CO_2_. Live cell imaging was performed with a 100× NA 1.46 objective and the following laser setting: sfGFP (excitation at 448 nm, emission was collected at 505–540 nm); mCherry (excitation at 561 nm, emission was collected at 580–610 nm); MitoTracker Deep Red (excitation at 633 nm, emission was collected at 655–710 nm). As lysosomes move fast and randomly, to avoid lysosome movement along Z-direction leading to imaging focus loss, gelatin was used to coat the glass bottom of the imaging dish to make cells attaching and spreading well at a XY-direction. To track the same population of lysosomes across time-lapse imaging, 0.5 μg/ml Nocodazole was added 15 minutes before neutralization to reduce lysosomal movement during neutralization and recovery.

To reproducibly define lysosome pH and the distance between mitochondria and lysosomes, we implemented an image analysis program in ImageJ. ROIs that contain single cell were manually selected using polygon tool and signals outside the ROIs were cleared to ensure the lysosome and mitochondria signal come from the same cell. To segment lysosomes, combined signal from both GFP and mCherry channels were used. We performed rolling ball background subtraction (radius = 3 pixels) followed by grey scale attribute filtering (operation=Opening attribute=Area minimum=4 connectivity=4) to the combined signal and generated the lysosome mask with auto local thresholding (Brensen method). ROIs for each separated lysosome or cluster of lysosomes were generated with “Analyze Particles” plugin. For each ROI, we calculated mean intensity of GFP and mCherry. The green-to-red ratio indicates the acidification of the lysosome. The mask for the mitochondria was generated by auto thresholding (Otsu method) the Mito Tracker signal. For each lysosome, the position of each pixel within the ROI was recorded, and for each pixel we determined the shortest distance to the mitochondria by looping through each point in the mitochondria mask with the Euclidean distance. The averaged shortest distance of all these lysosome pixels to the mitochondria is used to determine the distance between the lysosome and mitochondria. This effectively represents the distance between the center of lysosome to the mitochondria surface.

#### pH calibration

Yeast or mammalian cells were exposed to a potassium-phosphate buffer (50 mM potassium phosphate, 80 mM potassium chloride, 1 mM magnesium chloride) containing 5 μM nigericin (Sigma N7143) and 10 μM monensin (Sigma M5273) at pH 4.0 to pH 8.0 (for yeast cell, 2 mM DNP (2,4-dinitrophenol) was added in addition to nigericin and monensin). Cells were incubated in the buffers for 5 minutes before imaging to equilibrate extracellular pH.

#### RT-qPCR

For RT-qPCR assays, total RNA from mammalian cells was isolated with TRIzol LS agent. Each RNA sample (1 μg) was subjected to reverse transcription (Invitrogen SuperScript III One-Step RT-PCR System with Platinum Taq DNA Polymerase, Invitrogen 12574018), and then amplified by real-time PCR (Ssoadvanced Universal SYBR, Bio-Rad 1725271). The primers used for qPCR were shown in key resources table. The relative values of gene expression were calculated using the 2^−ΔΔCT^ method (PMID: 11846609) by comparing the cycle number for each sample with that for the untreated control. The results were normalized to the expression level of actin gene. All experiments have three independent replicates, and the mean was calculated for the figures.

#### RNA-seq data analysis and gene set enrichment

To quantify gene expression level, featureCounts v1.5.0-p3 was used to count the reads numbers mapped to each gene. And then FPKM of each gene was calculated based on the length of the gene and reads count mapped to this gene. FPKM, expected number of Fragments Per Kilobase of transcript sequence per Millions base pairs sequenced, considers the effect of sequencing depth and gene length for the reads count at the same time, and is currently the most commonly used method for estimating gene expression levels. We used clusterProfiler R package to test the statistical enrichment of differential expression genes in KEGG pathways.

### QUANTIFICATION AND STATISTICAL ANALYSIS

Experiments were repeated multiple times to confirm reproducibility. Image quantifications were done by batch processing without knowing the strain details. All quantifications are presented as the means ± standard error of mean (SEM), with each dot from one repeat. Data were analyzed with unpaired two-tailed t test: *, p<0.05; **, p<0.01; ***, p<0.001; ****, p<0.0001; ns, not significant. Statistical test for each figure was determined in Prism 9. “n” values are reported in the Table S1.

## Supplementary Material

Table S2

Table S1

Video S1

Video S2

Video S3

1

Supplemental information can be found online at https://doi.org/10.1016/j.molcel.2026.05.004.

## Figures and Tables

**Table:** KEY RESOURCES TABLE

REAGENT or RESOURCE	SOURCE	IDENTIFIER
Antibodies		
Pma1 antibody	Thermo Fisher	MA1–91567
GFP antibody	Sigma	SAB4301138
Flag antibody	Sigma	F3165
LC3 antibody	Cell Signaling Technology	mAb #12741
P62 antibody	Abcam	Ab109012
Chemicals, peptides, and recombinant proteins		
Quinacrine dihydrochloride	Sigma	Q3251
Calcofluor White	Sigma	18909–100 ML-F
Antimycin A	Sigma	A8674
Oligomycin	Sigma	75351
MitoTracker Deep Red FM	Invitrogen	M22426
NHiCl	VWR	BDH9208
Nocodazole	Sigma	M1404
X-Gal	Life Technologies	15520–018
SuperScript^™^ III One-Step RT-PCR System	Invitrogen	12574018
Click-iT EdU Alexa Fluor 488 HCS Assay	ThermoFisher Scientific	C10351
Senescence Detection Kit, SA-β-Gal kit	Abcam	ab65351
Lysosomal Acidic pH Detection Kit	Dojindo	L266
RNeasy Mini Kit	Qiagen	74104
Deposited data		
Raw data	This study	Mendeley Data: DOI: 10.17632/j99xc458mz.1
RNAseq raw data	This study	GEO: GSE292573
Experimental models: Organisms/strains		
*MATa his3Δ1 leu2Δ0 met15Δ0 ura3Δ0, TRP1::p404-GAL1-Tom70-HIS3*	this study	N/A
*MATa his3Δ1 leu2Δ0 met15Δ0 ura3Δ0, TRP1::p404-TDH3-preSu9-mCherry-NatMX*	this study	N/A
*MATa his3Δ1 leu2Δ0 met15Δ0 ura3Δ0, TRP1::p404-TDH3-preSu9-mCherry-NatMX; TRP1::pRS416-Z3EV-Tom70-URA3*	this study	N/A
*MATa his3Δ1 leu2Δ0 met15Δ0 ura3Δ0, TRP1::p404-CYC1-mCherry op-Fis1TM-KanMX*	this study	N/A
*MATa his3Δ1 leu2Δ0 met15Δ0 ura3Δ0, TRP1::p404-GAL1-VPS10-mCherry nanobody-HIS3*	this study	N/A
*MATa his3Δ1 leu2Δ0 met15Δ0 ura3Δ0, TRP1::p404-CYC1-mCherry op-Fis1TM-KanMX; TRP1::p404-GAL1-VPS10-mCherry nanobody-HIS3*	this study	N/A
*MATa his3Δ1 leu2Δ0 met15Δ0 ura3Δ0, TRP1::p404-CYC1-mCherry op-Fis1TM-KanMX; TRP1::p404-GAL1-VPS10-mCherry-HIS3*	this study	N/A
*MATa his3Δ1 leu2Δ0 met15Δ0 ura3Δ0, TRP1::p404-TDH3-mCherry-Scs2TM-KanMX; TRP1::p404-GAL1-VPS10-mCherry nanobody-HIS3*	this study	N/A
*MATa his3Δ1 leu2Δ0 met15Δ0 ura3Δ0, TRP1::p404-TDH3-sfCFP-mCherry-Fis1TM-KanMX*	this study	N/A
*MATa his3Δ1 leu2Δ0 met15Δ0 ura3Δ0, TRP1::p404-TDH3-sfCFP-mCherry-KanMX*	this study	N/A
*MATa his3Δ1 leu2Δ0 met15Δ0 ura3Δ0, TRP1:: p404-TDH3-sfCFP-mCherry-Fis1TM-KanMX; TRP1::p404-GAL1-VPS10-mCherry nanobody-HIS3*	this study	N/A
*MATa his3Δ1 leu2Δ0 met15Δ0 ura3Δ0, TRP1::p404-GAL1-Ypt7-HIS3*	this study	N/A
*MATa his3Δ1 leu2Δ0 met15Δ0 ura3Δ0, TRP1::p404-GAL1-Vam6-HIS3*	this study	N/A
*MATa his3Δ1 leu2Δ0 met15Δ0 ura3Δ0, TRP1::p404-GAL1-Ypt7-HIS3; TRP1::p404-GAL1-Vam6-HIS3-LEU2*	this study	N/A
*MATa his3Δ1 leu2Δ0 met15Δ0 ura3Δ0, ypt7Δ-HygMX*	this study	N/A
*MATa his3Δ1 leu2Δ0 met15Δ0 ura3Δ0, vam6Δ-HygMX*	this study	N/A
*MATa his3Δ1 leu2Δ0 met15Δ0 ura3Δ0, TRP1::p404-ADH-KanMx;pHO-NatMX-vSEP-yEmCherry*	this study	N/A
*MATa his3Δ1 leu2Δ0 met15Δ0 ura3Δ0, TRP1::p404-CYC1-mCherry-Fis1TM-KanMX; pHO-NatMX-vSEP-yEmCherry; p404-GAL1-Ypt7-HIS3; TRP1::p404-GAL1-Vam6-HIS3-LEU2; pHO-NatMX-vSEP-yEmCherry*	this study	N/A
*MATa his3Δ1 leu2Δ0 met15Δ0 ura3Δ0, TRP1::p404-CYC1-mCherry-Fis1TM-KanMX; pHO-NatMX-vSEP-yEmCherry;p404-GAL1-VPS10-mCherry nanobody-HIS3*	this study	N/A
*MATa his3Δ1 leu2Δ0 met15Δ0 ura3Δ0, TRP1::p404-CYC1-mCherry-Fis1TM-KanMX; pRS316-GFP-ATG8-URA3*	this study	N/A
*MATa his3Δ1 leu2Δ0 met15Δ0 ura3Δ0, TRP1::p404-CYC1-mCherry-Fis1TM-KanMX; TRP1::p404-GAL1-VPS10-mCherry nanobody-HIS3; pRS316-GFP-ATG8-URA3*	this study	N/A
*MATa his3Δ1 leu2Δ0 met15Δ0 ura3Δ0, Pma1-9aa-GFP-HIS3*	yeast GFP collection	N/A
*MATa his3Δ1 leu2Δ0 met15Δ0 ura3Δ0, Pma1-23aa-GFP-KanMX*	this study	N/A
*MATa his3Δ1 leu2Δ0 met15Δ0 ura3Δ0, Zrc1-GFP1–10-URA3; TRP1::p404-GAL1-Tom70-GFP11-HIS3*	this study	N/A
*MATa his3Δ1 leu2Δ0 met15Δ0 ura3Δ0, TRP1::p404-CYC1-mCherry op-Fis1TM-KanMX; ZRC1::p404-ZRC1-Zrc1-mCherry nanobody-HIS3*	this study	N/A
*MATa his3Δ1 leu2Δ0 met15Δ0 ura3Δ0, Vam6-GFP-HIS3*	yeast GFP collection	N/A
*MATa his3Δ1 leu2Δ0 met15Δ0 ura3Δ0, Zrc1-GFP-HIS*	yeast GFP collection	N/A
*MATa his3Δ1 leu2Δ0 met15Δ0 ura3Δ0, TRP1::p404-GAL1-Tom70-GFP-HIS3*	this study	N/A
*MATa his3Δ1 leu2Δ0 met15Δ0 ura3Δ0, Vma1-GFP-HIS3*	yeast GFP collection	N/A
*MATa his3Δ1 leu2Δ0 met15Δ0 ura3Δ0, Vma2-GFP-HIS3*	yeast GFP collection	N/A
*MATa his3Δ1 leu2Δ0 met15Δ0 ura3Δ0, Vma5-GFP-HIS3*	yeast GFP collection	N/A
*MATa his3Δ1 leu2Δ0 met15Δ0 ura3Δ0, Vma8-GFP-HIS3*	yeast GFP collection	N/A
*MATa his3Δ1 leu2Δ0 met15Δ0 ura3Δ0, Vma4-GFP-HIS3*	yeast GFP collection	N/A
*MATa his3Δ1 leu2Δ0 met15Δ0 ura3Δ0, Vma7-GFP-HIS3*	yeast GFP collection	N/A
*MATa his3Δ1 leu2Δ0 met15Δ0 ura3Δ0, Vma10-GFP-HIS3*	yeast GFP collection	N/A
*MATa his3Δ1 leu2Δ0 met15Δ0 ura3Δ0, Vma13-GFP-HIS3*	yeast GFP collection	N/A
*MATa his3Δ1 leu2Δ0 met15Δ0 ura3Δ0, Vph1-GFP-HIS3*	yeast GFP collection	N/A
*MATa his3Δ1 leu2Δ0 met15Δ0 ura3Δ0, Vma16-GFP-HIS3*	yeast GFP collection	N/A
*MATa his3Δ1 leu2Δ0 met15Δ0 ura3Δ0, TRP1::p404-TDH3-preSU9-GFP-NatMX*	this study	N/A
*MATa his3Δ1 leu2Δ0 met15Δ0 ura3Δ0, Qcr7-GFP-HIS3*	yeast GFP collection	N/A
*MATa his3Δ1 leu2Δ0 met15Δ0 ura3Δ0, bni1A-KanMX; TRP1::p404-TDH3-preSu9-mCherry-NatMX*	this study	N/A
*MATa his3Δ1 leu2Δ0 met15Δ0 ura3Δ0, bni1Δ-KanMX; Tom70-GFP-HIS3*	this study	N/A
*MATa his3Δ1 leu2Δ0 met15Δ0 ura3Δ0, bni1::bni1#1-HIS3*	David Pellman lab	N/A
*MATa his3Δ1 leu2Δ0 met15Δ0 ura3Δ0, Δypt11-KanMX; pGAL1-mmr1-HIS3;TRP1::p405-TDH3-pOLI-HcRed-LEU2*	this study	N/A
*MATa his3Δ1 leu2Δ0 met15Δ0 ura3Δ0, Mob2-GFP-HIS3*	yeast GFP collection	N/A
*MATa his3Δ1 leu2Δ0 met15Δ0 ura3Δ0, aip5Δ-KanMX*	yeast knockout collection	N/A
*MATa his3Δ1 leu2Δ0 met15Δ0 ura3Δ0, cla4Δ-KanMX*	yeast knockout collection	N/A
*MATa his3Δ1 leu2Δ0 met15Δ0 ura3Δ0, lrg1Δ-KanMX*	yeast knockout collection	N/A
*MATa his3Δ1 leu2Δ0 met15Δ0 ura3Δ0, skm1Δ-KanMX*	yeast knockout collection	N/A
*MATa his3Δ1 leu2Δ0 met15Δ0 ura3Δ0, ssd1Δ-KanMX*	yeast knockout collection	N/A
*MATa his3Δ1 leu2Δ0 met15Δ0 ura3Δ0, ste20Δ-KanMX*	yeast knockout collection	N/A
*MATa his3Δ1 leu2Δ0 met15Δ0 ura3Δ0, wsc2Δ-KanMX*	yeast knockout collection	N/A
*MATa his3Δ1 leu2Δ0 met15Δ0 ura3Δ0, cbk1ts-KanMX*	Charles Boone lab	N/A
*MATa his3Δ1 leu2Δ0 met15Δ0 ura3Δ0, cdc42ts-KanMX*	Charles Boone lab	N/A
*MATa his3Δ1 leu2Δ0 met15Δ0 ura3Δ0, mob1ts-KanMX*	Charles Boone lab	N/A
*MATa his3Δ1 leu2Δ0 met15Δ0 ura3Δ0, pkc1ts-KanMX*	Charles Boone lab	N/A
*MATa his3Δ1 leu2Δ0 met15Δ0 ura3Δ0, mob2ts-KanMX*	Charles Boone lab	N/A
*MATa his3Δ1 leu2Δ0 met15Δ0 ura3Δ0, Mob2-Flag-URA3*	this study	N/A
*MATa his3Δ1 leu2Δ0 met15Δ0 ura3Δ0, Vam6-Flag-URA3*	this study	N/A
*MATa his3Δ1 leu2Δ0 met15Δ0 ura3Δ0, mob2ts-KanMX, Vam6-Flag-URA3*	this study	N/A
*MATa his3Δ1 leu2Δ0 met15Δ0 ura3Δ0, mob2ts-KanMX, Vam6-GFP-HIS3*	this study	N/A
*MATa his3Δ1 leu2Δ0 met15Δ0 ura3Δ0, Vam6-GFP-HIS3, Mob2-Flag-KanMX*	this study	N/A
Oligonucleotides		
*See* Table S2 *for primers used in this study*	This study	N/A
Recombinant DNA		
*p404-GAL1-Tom70-HIS3*	this study	N/A
*p404-TDH3-preSu9-mCherry-NatMX*	this study	N/A
*pRS416-Z3EV-Tom70-URA3*	this study	N/A
*p404-GAL1-VPS10-mCherry nanobody-HIS3*	this study	N/A
*p404-GAL1-VPS10-mCherry-HIS3*	this study	N/A
*p404-TDH3-sfGFP-mCherry-Fis1TM-KanMX*	this study	N/A
*p404-TDH3-sfGFP-mCherry-KanMX*	this study	N/A
*p404-GAL1-Ypt7-HIS3*	this study	N/A
*p404-GAL1-Vam6-HIS3*	this study	N/A
*p404-GAL1-Vam6-HIS3-LEU2*	this study	N/A
*p404-TDH3-mCherry-Scs2TM-KanMX*	this study	N/A
*p404-CYC1-mCherry op-Fis1TM-KanMX*	this study	N/A
*p404-GAL1-Tom70-GFP11-HIS3*	this study	N/A
*p404-GAL1-Tom70-GFP-HIS3*	this study	N/A
*p404-ZRC1-Zrc1-mCherry nanobody-HIS3*	this study	N/A
*pCMV-sfGFP-LAMP1-mCherry (pHLARE)*	Diane L. Barber lab	N/A
*pTET-Tom20N mCherry nanobody & pCMV-sfGFP-LAMP1-mCherry (pHLARE)*	this study	N/A
*pHO-Nat-vSEP-yEmCherry*	Gottschling lab	N/A
Software and algorithms		
ImageJ	NIH	RRID:SCR_003070
GraphPad Prism 9	GraphPad	RRID:SCR_002798

## Highlights

- Mitochondria supply protons to lysosomes/vacuoles through membrane contact sites
- Loss of mitochondria-lysosome/vacuole contacts drives de-acidification in aging cells
- Asymmetric division restores Mito-Vac contacts in rejuvenated daughter cells
- Restoring Mito-Lyso contacts in senescent cells rescues autophagy and inhibits SASP
