# Supplementary material for: Mitochondria-lysosome coupling contributes to lysosome acidification and aging
Source: Mol Cell. Author manuscript; Available in PMC 2026 Jun 14. (PMC13263124; doi:10.1016/j.molcel.2026.05.004)
Supplement: 1 [file NIHMS2182381-supplement-1.pdf]

Molecular Cell, Volume *86*

Supplemental information

Mitochondria-lysosome coupling contributes to lysosome acidification and aging

Qingqing Liu, Seungmin Yoo, Zhixin A. Zhang, Liying Li, Hetian Su, Lingraj Vannur, Alexandra C. Wooldredge, Jun-Wei B. Hughes, Pierre-Yves Desprez, Nan Hao, Gordon Lithgow, Julie K. Andersen, Malene Hansen, Judith Campisi, and Chuankai Zhou

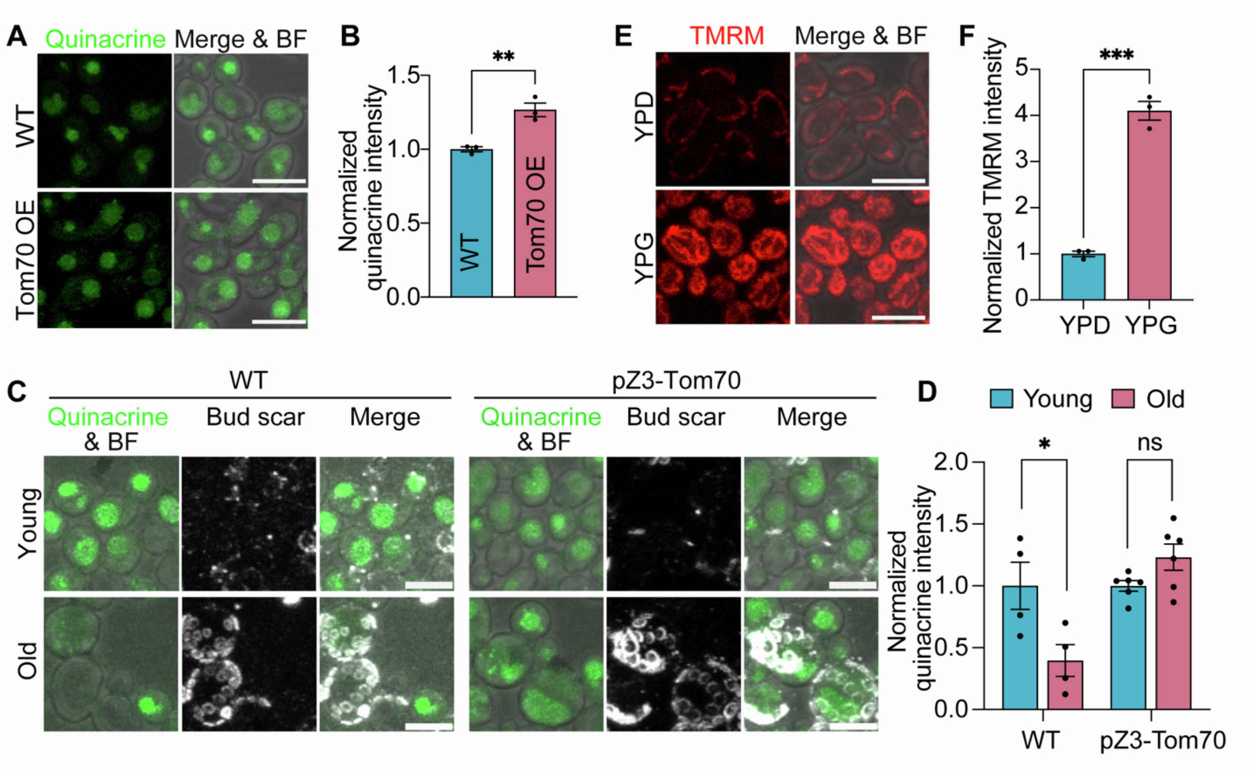

Figure S1 Mitochondria promote vacuolar acidification, related to Figure 1.

(A, B) Representative images and quantification of quinacrine staining in young cells of wild-type (WT) and Tom70 overexpression (Tom70 OE). Tom70 was overexpressed from pGAL promoter. All strains were cultured in the YP-Galactose medium for comparison. BF, bright field.

(C, D) Representative images and quantification of quinacrine staining in young and old cells of WT and Tom70 OE (under pZ3 promoter and induced by β-estradiol). All strains here were cultured in the YPD medium for comparison with the strains cultured in YP-Galactose medium in Figure 1B and 1C to exclude the effect of carbon source difference. Bud scar was stained with Wheat Germ Agglutinin, Alexa Fluor 594 to indicate the cell age. BF, Bright field.

(E, F) Representative images and quantification of mitochondrial membrane potential shown with TMRM staining of cells cultured in YPD and YPG media.

Bar graphs are means ± SEM. Data were analyzed with unpaired two-tailed t test: *, $p<0.05$; **, $p<0.01$; ***, $p<0.001$; ns, not significant. Scale bars: 5 μm.

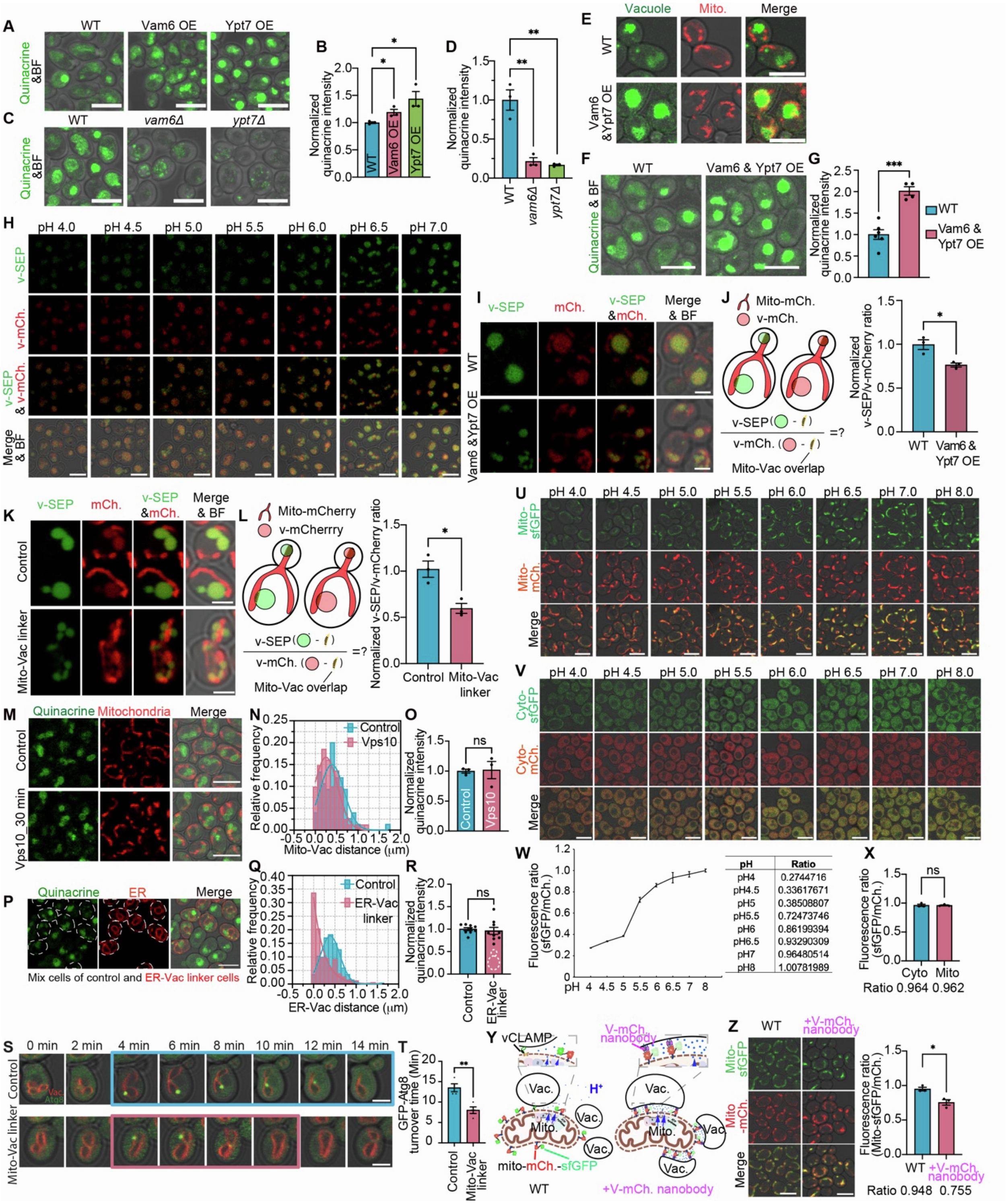

Figure S2 Additional data and controls that mitochondria-vacuole contact affects vacuolar acidification, related to Figure 2.

(A-D) Representative images and quantification of quinacrine staining in WT, Vam6 overexpression (Vam6 OE), Ypt7 overexpression (Ypt7 OE) cells, as well as their knockout.

(E) Representative images showing that Vam6 & Ypt7 double overexpression (Vam6&Ypt7 OE) bridged mitochondria and vacuoles together. Vacuole was shown by quinacrine staining. Mito, mitochondria, shown by mCherry-Fis1tm.

(F and G) Representative images and quantification of quinacrine staining in WT and Vam6 & Ypt7 OE cells.

(H) Representative images showing the fluorescence intensity change of v-SEP in different pH buffer. mCh., mCherry.

(I-J) Representative images and quantification of v-SEP/v-mCherry ratio change in WT and Vam6&Ypt7 OE cells. 'v-' denotes vacuole localization, following the nomenclature used in the original study that reported this reporter [71]. Both Mito-mCherry and v-mCherry were used to label mitochondria and vacuoles, respectively, in the same cells. The Mito-Vac overlap region was excluded when quantifying v-SEP and v-mCherry intensities (also applied in Figure S2L). Quantification was performed on more than 50 cells per group. Scale bars, 2 μM.

(K-L) Representative images and quantification of v-SEP/v-mCherry ratio change in control and Mito-Vac linker expression cells. Quantification was performed on more than 50 cells per group. Scale bar, 2 μM.

(M-O) Representative images (M) and quantification (O) of quinacrine staining and Mito-Vac distance (N) in WT control cells and cells with 30 minutes Gal-induced expression of Vps10 (Vps10 cells). This is a control for Vps10-mCherry nanobody used in the Mito-Vac linker.

(P) Representative images of quinacrine staining in control and ER-Vac linker expressing cells. The engineered ER-Vac linker is made of mCherry-Scs2 on ER and Vps10-mCherry nanobody on vacuole. To avoid quinacrine staining variation, control and ER-Vac linker-containing cells were mixed in a 1:1 ratio and stained together. ER-Vac linker-containing cells (circled by dashed line) contain ER-mCherry as a marker.

(Q) Quantification of the distance between ER and vacuole in control and ER-Vac linker expressing cells.

(R) Quantification of quinacrine intensity in control and ER-Vac linker expressing cells.

(S, T) Timelapse images and quantification showing the turnover of GFP-Atg8 puncta during starvation in WT control cells and Mito-Vac linker expressing cells. The boxed images show the

formation and turnover of GFP-Atg8 puncta, indicating autophagic flux, with noticeably faster turnover in cells expressing the Mito-Vac linker. Scale bar: 2 μm. Data from four biological repeats (each with 7-10 cells) were analyzed.

(U-W) Representative images and quantification showing pH calibration of the pH sensor on the mitochondrial surface or in the cytosol. The ratiometric biosensor consists of pH-sensitive superfolder GFP (sfGFP) and relatively pH-insensitive mCherry. sfGFP exhibits lower signal at lower pH. The Mito-biosensor is anchored to mitochondrial surface by tagging it to the N-terminus of Fis1tm. Cells were incubated in the buffer of different pH with nigericin, monensin, and DNP to equalize proton across membranes. More than 400 cells from three biological repeats were quantified for each pH.

(X) Fluorescence ratios of sfGFP-mCherry biosensor in the cytosol and on the mitochondrial surface of wild-type control cells. Cyto, biosensor expressed in the cytosol. Mito, biosensor anchored on mitochondrial surface. No significant difference observed, likely because the protons diffuse quickly and do not concentrate locally on the mitochondrial surface (see 'WT control' in Figure S2Y).

(Y) Schematic illustration of the distribution and signal of sfGFP-mCherry-Fis1tm biosensor on the mitochondrial surface with or without V-mCh nanobody expression. The ratiometric biosensor is consisted of pH-sensitive superfolder GFP (sfGFP) and pH-insensitive mCherry. mCherry nanobody is anchored on vacuole surface by Vps10 (V-mCh nanobody). In wild-type control cells, Mito-Vac contacts (vCLAMPs) are dynamic, and protons released from mitochondria rapidly diffuse or are absorbed outside these contact sites; as a result, the sfGFP–mCherry heterodimer—distributed evenly across the mitochondrial surface, mostly outside of organellar contact regions—exhibits an average sfGFP/mCherry ratio similar to that of the bulk cytosol. In contrast, in cells expressing V-mCh nanobody, which binds mCherry and concentrates the sfGFP-mCherry heterodimer at the Mito-Vac contact site, the sfGFP/mCherry ratio significantly decreased.

(Z) Representative images and ratio quantification of sfGFP-mCherry-Fis1tm biosensor on the mitochondrial surface in wild-type control cells and the cells expressing the V-mCh nanobody to recruit biosensors to the Mito-Vac contacts.

Bar graphs and line graphs are means ± SEM. Data were analyzed with unpaired two-tailed t test: *, $p<0.05$; **, $p<0.01$; ***, $p<0.001$; ns, not significant. Scale bars, 5 μm if not mentioned.

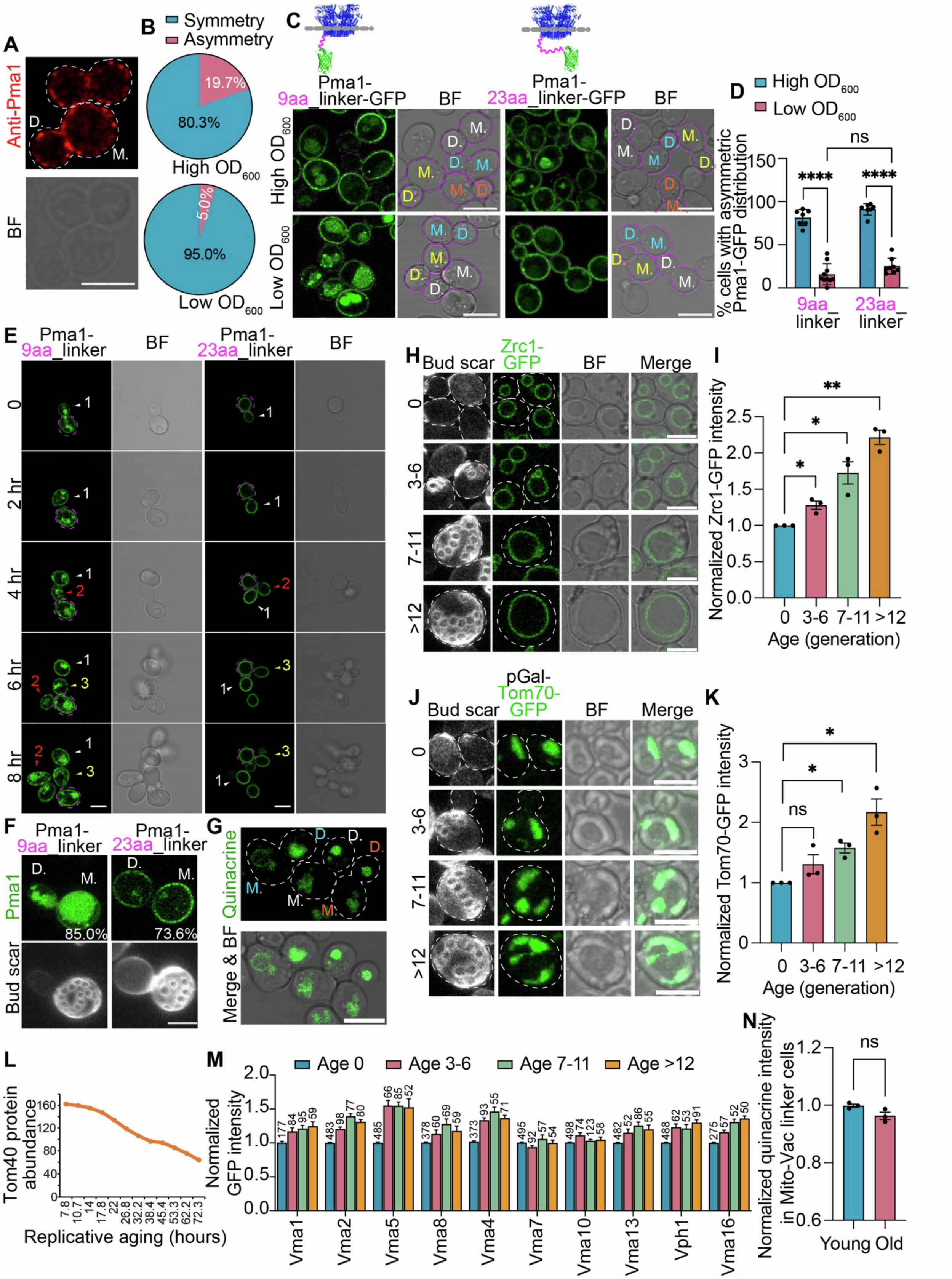
A
Anti-Pma1
D.
M.
BF
B
Symmetry
Asymmetry
19.7%
80.3%
High OD600
5.0%
95.0%
Low OD600
C
Pma1-
9aa_linker-GFP
BF
Pma1-
23aa_linker-GFP
BF
High OD600
Low OD600
D
High OD600
Low OD600
% cells with asymmetric Pma1-GFP distribution
ns
****
****
150
100
50
0
9aa_ linker
23aa_ linker
E
Pma1-
9aa_linker
BF
Pma1-
23aa_linker
BF
0
2 hr
4 hr
6 hr
8 hr
F
Pma1-
9aa_linker
Pma1-
23aa_linker
Pma1
Bud scar
85.0%
73.6%
G
Quinacrine
Merge & BF
H
Bud scar
Zrc1-
GFP
BF
Merge
0
3-6
7-11
>12
I
Normalized Zrc1-GFP intensity
Age (generation)
J
Bud scar
pGal-
Tom70-
GFP
BF
Merge
K
Normalized Tom70-GFP intensity
Age (generation)
L
Tom40 protein abundance
Replicative aging (hours)
M
Age 0
Age 3-6
Age 7-11
Age >12
Normalized GFP intensity
Vma1
Vma2
Vma5
Vma8
Vma4
Vma7
Vma10
Vma13
Vph1
Vma16
N
Normalized quinacrine intensity in Mito-Vac linker cells
ns
Young
Old

Figure S3 Lack of Pma1 accumulation in mother cells during aging and additional data related to age-associated reduction of mitochondria-vacuole contacts, related to Figure 3.

(A, B) Representative images and quantification of immunofluorescence staining of endogenous Pma1 by antibody. Pie charts show the percentage of cells with symmetric and asymmetric distribution of endogenous Pma1 in cells experienced different cell density after overnight culture before refreshing in next day (low OD_{600}:~0.5 after overnight culture; high OD_{600}:>2 after overnight culture, same for other figures. See details in Method). D., daughter cell; M. mother cell.

(C, D) Representative images and quantification of the distribution of Pma1-(9aa_linker)-GFP and Pma1-(23aa_linker)-GFP in cells experienced different culture density after overnight culture before refreshing. Pma1-(9aa_linker)-GFP is a strain from GFP library and has 9 amino acid (aa) linker, while Pma1-(23aa_linker)-GFP has 23 amino acid linker between Pma1 and GFP open reading frame. Notably, the short linker caused significant mislocalization of Pma1 to the vacuole similar to the one observed in previous study[35,36], whereas the long linker allowed Pma1 to localize properly to the plasma membrane. BF, bright field. M., mother cell. D., daughter cell (bud).

(E) Time lapse images showing the protein level and distribution of Pma1-(9aa_linker)-GFP and Pma1-(23aa_linker)-GFP. The numbers and arrows indicate the generations of daughter cells produced by the mother cell (circled by dashed lines).

(F) Representative images and quantification showing the protein distribution of Pma1-(9aa_linker)-GFP and Pma1-(23aa_linker)-GFP in old cells. The inserted numbers showing the percentage of cells with this phenotype were quantified from 80 and 91 of old cells for Pma1-(9aa_linker)-GFP and Pma1-(23aa_linker)-GFP from three biological repeats. Notably, both (E) and (F) show that the aging mother cells do not accumulate high level of Pma1 on the plasma membrane, albeit significant amount of proteins were degraded in vacuole for the short linker Pma1-GFP. M., mother cell. D., daughter cell (bud). The inserted numbers are percentage of cells showing the representative phenotypes.

(G) Representative images showing the asymmetry in vacuole pH between mother and daughter cells kept at low mid-log density. M., mother cell. D., daughter cell (bud).

(H-K) Representative images and quantification of endogenous expression level of Zrc1-GFP and the pGal-induced expression level of Tom70-GFP in cells of different ages. This is a control for

the reduction of split-GFP signal in old cells of Figure 3B and 3C as Zrc1 protein or Gal induction of Tom70 did not decline during aging.

(L) Quantification of the Tom40 protein level during aging (proteomics data from Janssens et al., 2015 *eLife*[38]).

(M) Protein abundance of different V-ATPase subunits during yeast replicative aging quantified with strains from yeast GFP library. The numbers labeled on the top of bars indicate the amount of cells quantified.

(N) Quantification of quinacrine intensity in young and old cells expressing low level of Mito-Vac linker used in RLS experiment of Figure 3I (pZrc1-Zrc1-mCherry nanobody and mCherry-Fis1tm).

Bar graphs are means ± SEM. Data were analyzed with unpaired two-tailed t test: *, $p<0.05$; **, $p<0.01$; ns, not significant. Scale bars, 5 μm.

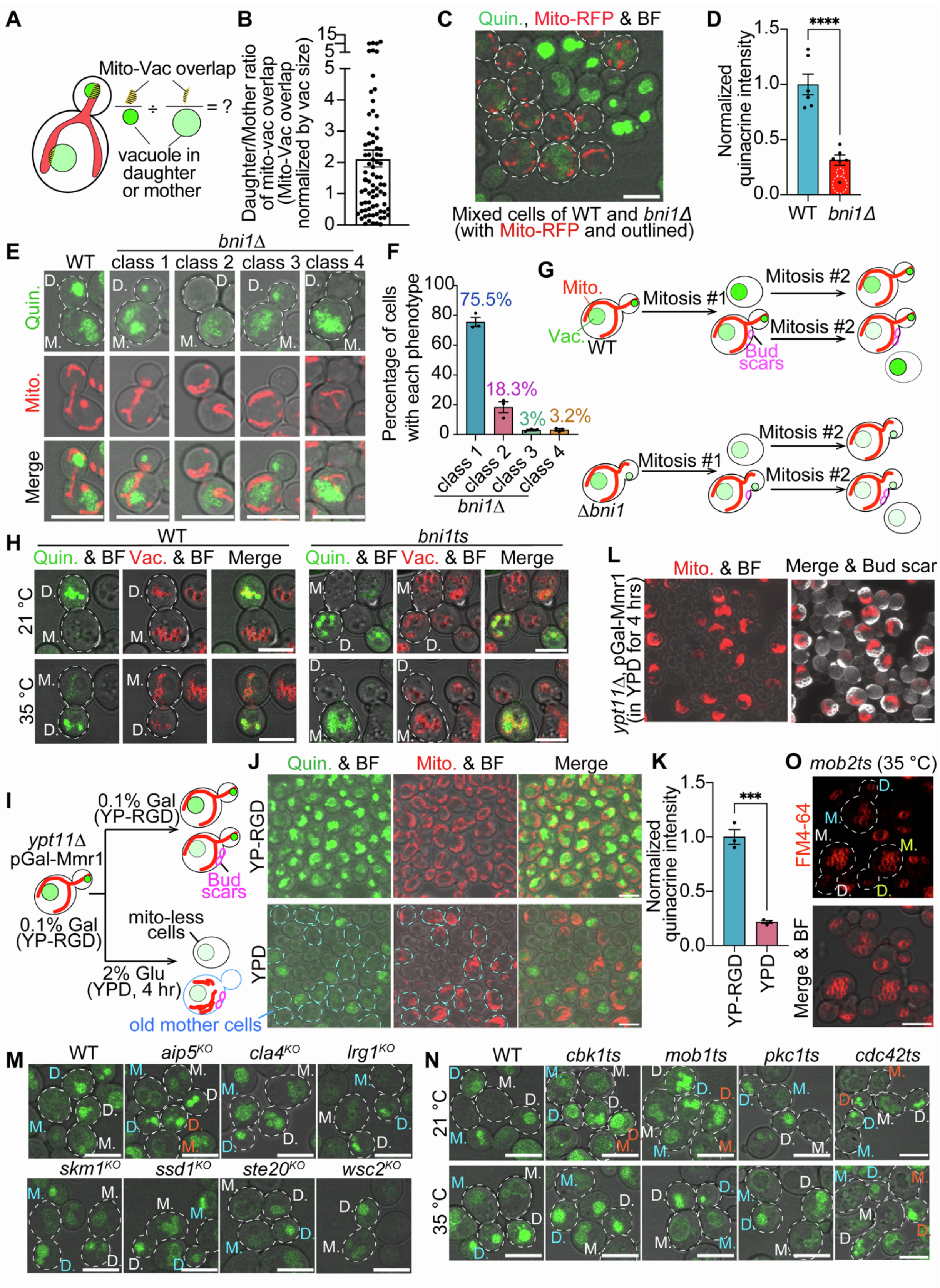

A
Mito-Vac overlap
vacuole in daughter or mother
B
Daughter/Mother ratio of mito-vac overlap (Mito-Vac overlap normalized by vac size)
C
Quin., Mito-RFP & BF
Mixed cells of WT and bni1Δ (with Mito-RFP and outlined)
D
Normalized quinacrine intensity
****
WT
bni1Δ
E
WT
bni1Δ
class 1 class 2 class 3 class 4
Quin.
Mito.
Merge
F
Percentage of cells with each phenotype
75.5%
18.3%
3%
3.2%
class 1 class 2 class 3 class 4
bni1Δ
G
Mito.
Vac.
WT
Mitosis #1
Mitosis #2
Bud scars
Δbni1
H
WT
bni1ts
Quin. & BF Vac. & BF Merge
21 °C
35 °C
I
ypt11Δ pGal-Mmr1
0.1% Gal (YP-RGD)
Bud scars
mito-less cells
2% Glu (YPD, 4 hr)
old mother cells
J
Quin. & BF
Mito. & BF
Merge
YP-RGD
YPD
K
Normalized quinacrine intensity
***
YP-RGD
YPD
L
Mito. & BF
Merge & Bud scar
ypt11Δ, pGal-Mmr1 (in YPD for 4 hrs)
O
mob2ts (35 °C)
FM4-64
Merge & BF
M
WT
aip5KO
cla4KO
lrg1KO
skm1KO
ssd1KO
ste20KO
wsc2KO
N
WT
cbk1ts
mob1ts
pkc1ts
cdc42ts
21 °C
35 °C

Figure S4 Additional data and controls for vacuole re-acidification in daughter cells, related to Figure 4.

(A, B) The schematics and quantification of the Mito-Vac overlap in mother cell and bud. The mitochondria overlapped with vacuole was normalized by vacuole size in the corresponding cell. Mitochondria were visualized with Qcr7-GFP and vacuole were stained with FM4-64. This normalized Mito-Vac overlap of the daughter cell was then compared with its mother cell to get the Daughter/Mother ratio in (B).

(C, D) Representative images and quantification of quinacrine staining in WT and *bni1Δ* (outlined by white dash line and expressing mito-RFP) cells. BF, bright field. Quin., quinacrine.

(E, F) Representative images and quantification of the inheritance of mitochondria and vacuoles in WT and *bni1Δ* cells. In WT cells, the actin cables converge at the bud tips, which provide the track for mitochondria and vacuoles to contact each other at the bud tips. The actin cable nucleation at the bud tips is abolished in *bni1Δ* cells, thereby impairing the bud-tip-oriented organelle inheritance: Mitochondria and vacuoles failed to be pulled toward the bud tip and instead accumulated near the bud neck, with the distance between the two organelles being greater in *bni1Δ* cells (class 1) compared to WT cells; mitochondria or vacuoles were not inherited by the buds simultaneously, resulting in mitochondria only or vacuole only buds (*bni1Δ* class 2 or class 3); or both organelles inheritance was completely blocked (*bni1Δ* class 4). M., mother cell. D., daughter cell (bud).

(G) Illustration for the population-level difference in the quinacrine staining among WT and *bni1*Δ cells. Due to the defect of vacuole re-acidification in *bni1*Δ daughter cells, when they mature into mother cells, these *bni1*Δ mother cells also lack vacuolar acidification in Figure S4C.

(H) Representative images of quinacrine staining in WT and *bni1ts* mutant with vacuole marker FM4-64. This is a control of quinacrine staining in Figure 4D that the loss of quinacrine staining in the daughter cells is not due to vacuole inheritance defects in *bni1ts* cells. M., mother cell. D., daughter cell (bud).

(I-K) Schematic diagram, representative images, and quantification of the vacuolar acidification in cells lacking mitochondrial inheritance. Media switch from balanced medium (YP-RGD contains raffinose, galactose, and dextrose) to YPD for 4 hrs to deplete Mmr1 and inhibit mitochondria inheritance in the presence of *ypt11*Δ. YPD, which contains 2% dextrose/glucose

but lacks galactose, inhibits the expression of pGal-Mmr1. After being cultured in YPD for 4 hours, most of the daughter cells failed to inherit mitochondria. The mother cells which retained mitochondria (blue outline) gradually lost vacuolar acidification due to replicative aging (see Figure S4L). BF, bright field.

(L) Calcofluor white staining of *ypt11Δ pGal-MMR1* cells after switch from YP-RGD to YPD medium for 4 hrs. Note that the mother cells with mitochondria are replicative old which caused vacuole de-acidification in Figure S4J. BF, bright field.

(M, N) Targeted screening among asymmetrically localized proteins for potential factors involved in the regulation of vacuole pH rejuvenation. A list of daughter cell-localized proteins previously implicated in asymmetric cell division was systematically knocked down or knockout to test their impact on the acidification of the vacuole within the daughter cell. The knockout/knockdown of potential regulator would abolish the asymmetric pattern of vacuole acidification between mother and daughter cell. Representative images showing the quinacrine staining in the gene knockout (M) and knockdown (N) mutated cells. Note that among these proteins only the *mob2ts* prevents the re-acidification of the vacuole in the daughter cells (See Figure 4K-L). ts, temperature sensitive. M., mother cell. D., daughter cell (bud).

(O) FM4-64 staining showing the vacuole inheritance in *mob2ts* cells at restrictive temperature (35°C). BF, bright field. M., mother cell. D., daughter cell (bud).

Bar graphs are means ± SEM. Data were analyzed with unpaired two-tailed t test: ***, $p<0.001$; ****, $p<0.0001$. Scale bars, 5 μm.

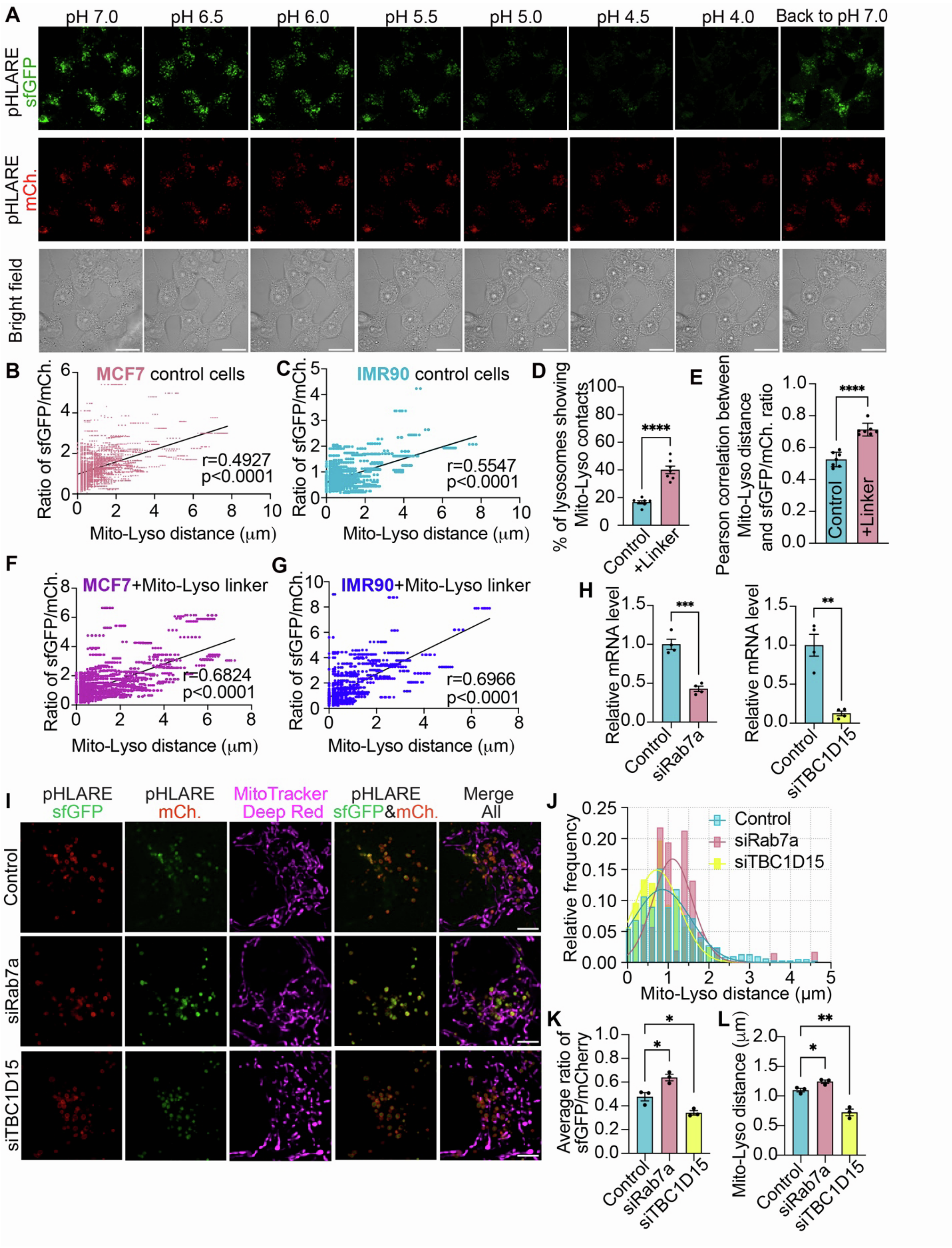

Figure S5 Additional data and controls for pHLARE and lysosome acidification under

different conditions, related to Figure 5.

(A) The pHLARE responses to the pH change in lysosomal lumen. The acid-sensitive sfGFP proportionally loses its fluorescence during the acidification of the lysosomal lumen. In cells treated with protonophore nigericin, the pH-buffered medium controls the pH of lysosomal lumen and affects the intensity of sfGFP. The fluorescence decline of sfGFP in low pH buffer is not due to laser bleaching during imaging, as the fluorescence recovered immediately after increasing the medium pH. Scale bar, 20 μm.

(B, C) The correlation of Mito-Lyso distance and lysosomal acidification in MCF7 and early passage IMR90 (pre-senescent) cells. Pearson correlation r and p value (two-tailed) are shown in the figure. The contact between mitochondria and lysosome is frequent and dynamic, typically lasting for 1-2 minutes before dissociating (with some mitochondria–lysosome contacts persist >5 minutes)[28,29]. In our re-acidification assay (Figure 5H), NH_4Cl-neutralized lysosomes required approximately 2–5 minutes to regain acidity, with lysosomes in stable contact with mitochondria acidifying more quickly than those farther away. This 2–5-minute time frame indicates that one or two cycles of contact and detachment between mitochondria and lysosomes are sufficient to re-acidify even fully neutralized lysosomes. Because lysosomes in live cells are never completely neutralized, these frequent and dynamic contacts with mitochondria appear sufficient for maintaining an acidic pH. This dynamic nature of Mito-Lyso contact and lysosomal acidification likely contributes to the heterogeneity of lysosomal acidity across the cell and explains why some lysosomes' acidification status does not always correlate with their contact to mitochondria. Lysosomes observed in contact with mitochondria during imaging may already be fully acidified, having spent 1-2 minutes at the mitochondrial surface, or they may have recently arrived and initiated their acidification process. Consistent with this, when organelle contacts were strengthened using a Mito-Lyso linker, we observed a stronger correlation between Mito-Lyso distance and lysosomal acidification status, as well as an enhanced lysosomal acidification (F, G).

(D) Percentage of lysosomes showing stable mitochondria-lysosome contacts in control and Mito-Lyso linker-expressing (Linker) HeLa cells. Bar graphs are means ± SEM. Data from seven biological repeats were analyzed with unpaired two-tailed t test: ****, $p<0.0001$.

(E) Pearson correlation values between Mito-Lyso distance and lysosome acidification (sfGFP/mCherry ratio) per cell were quantified and averaged for control and Mito-Lyso linker-

expressing (Linker) HeLa cells. Bar graphs are means ± SEM. Data from seven biological repeats were analyzed with unpaired two-tailed t test: ****, $p<0.0001$.

(F, G) The correlation of Mito-Lyso distance and lysosomal acidification in MCF7 and early passage IMR90 (pre-senescent) cells with Mito-Lyso linker. Pearson correlation r and p value (two-tailed) are shown in the figure.

(H) Quantification of Rab7a and TBC1D15 mRNA level after RNAi. **, $p<0.01$; ***, $p<0.001$.

(I) Representative images showing the changes of Mito-Lyso contact and lysosomal acidification upon RAB7a and TBC1D15 knockdown. Scale bar, 10 µm.

(J) Histogram showing the distribution frequency of Mito–Lyso contacts in control cells and in cells with Rab7a or TBC1D15 knockdown.

(K, L) Quantification of Mito-Lyso distance per cell (L) and lysosomal pH measurements obtained from more than 1000 lysosomes per group (K). Data are presented as mean ± SEM. P values determined by unpaired two-tailed Student’s t-test: *, $p<0.05$; **, $p<0.01$.

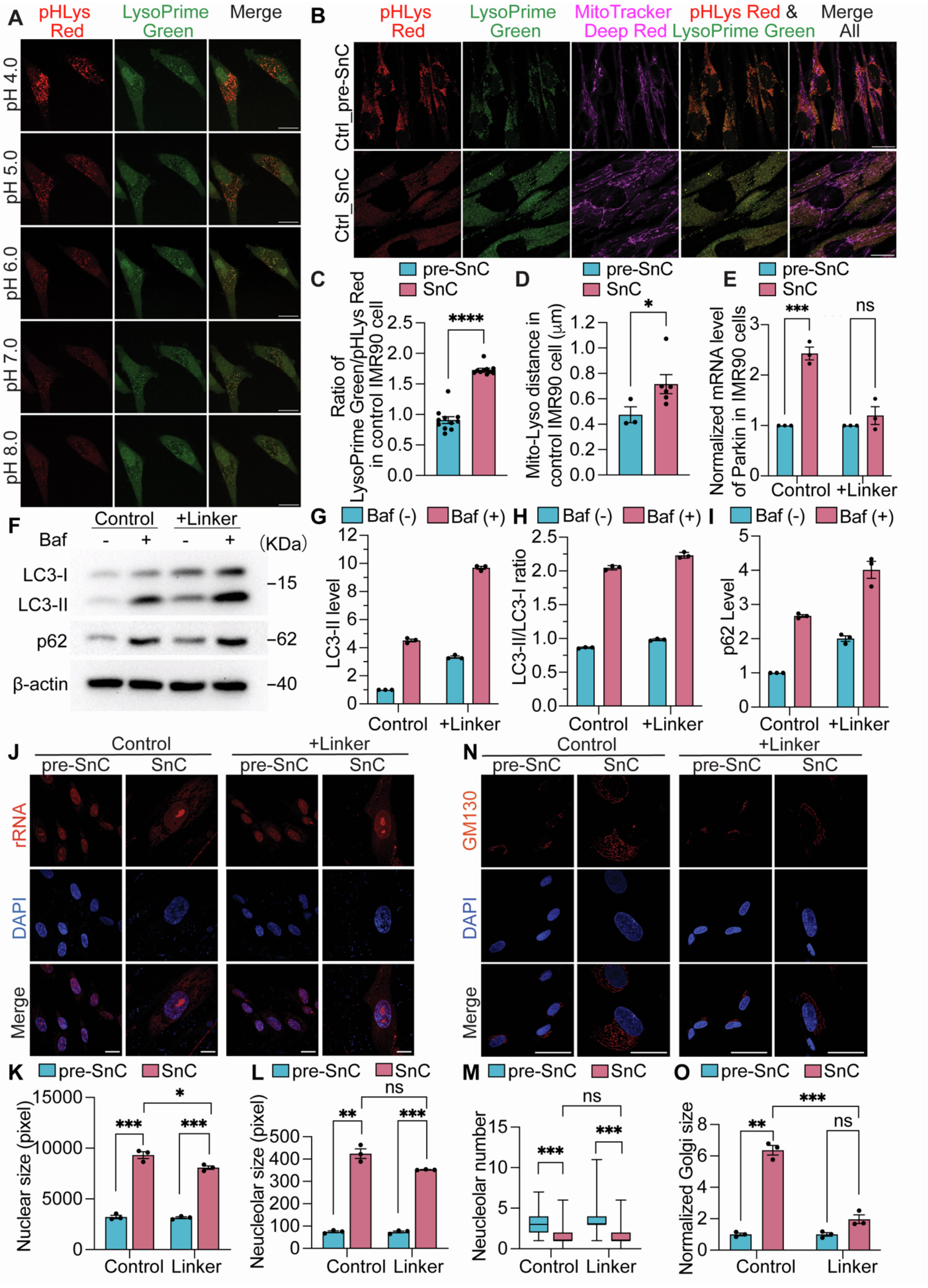

A
pHLys Red
LysoPrime Green
Merge
pH 4.0
pH 5.0
pH 6.0
pH 7.0
pH 8.0
B
pHLys Red
LysoPrime Green
MitoTracker Deep Red
pHLys Red & LysoPrime Green
Merge All
Ctrl_pre-SnC
Ctrl_SnC
C
pre-SnC
SnC
Ratio of LysoPrime Green/pHLys Red in control IMR90 cell
****
D
Mito-Lyso distance in control IMR90 cell (μm)
*
E
Normalized mRNA level of Parkin in IMR90 cells
***
ns
Control
+Linker
F
Control
+Linker
Baf
(KDa)
LC3-I
LC3-II
p62
β-actin
G
Baf (-)
Baf (+)
LC3-II level
H
LC3-II/LC3-I ratio
I
p62 Level
J
rRNA
DAPI
Merge
N
GM130
K
Nuclear size (pixel)
L
Neucleolar size (pixel)
M
Neucleolar number
O
Normalized Golgi size
Linker

221

Figure S6 Additional data and controls for lysosome acidification/function and other organelle changes in human senescent cells, related to Figure 6.

(A) Representative images from pH sensitivity test of pH-sensitive dye (pHLys Red) and pH-insensitive dye (LysoPrime Green) in IMR90 cells. Scale bar, 20 μm.

(B-D) Representative images and quantifications showing lysosome acidification (B, C) and Mito-Lyso distance (D) in wild-type control pre-senescent and senescent IMR90 cells. Lysosomes were stained with pH-sensitive dye (pHLys Red) and pH-insensitive dye (LysoPrime Green). The increased LysoPrime Green to pHLys Red ratio in (C), resulting from the loss of pHLys Red fluorescence in senescent IMR90 cells, indicates a defect in lysosome acidification, consistent with previous reports. Mitochondria were stained with MitoTacker Deep Red FM. Scale bar, 20 μm.

(E) Quantification of RT-qPCR results showing changes in Parkin mRNA level in IMR90 pre-senescent and senescent cells with or without Mito-Lyso linker expression. Control, wild-type IMR90 cells. +Linker, Mito-Lyso linker-expressing IMR90 cells. Same for other figures.

(F-I) Representative immunoblot and quantification of LC3-I, LC3-II, and p62 levels with/without the presence of bafilomycin A (Baf) in the control and Mito-Lyso linker expressing pre-senescent IMR90 cells. β-actin is used as loading control. Bar graphs are means ± SEM from three biological repeats.

(J) BioTracker rRNA Probe and DAPI staining of nucleolus and nucleus in pre-senescent and senescent cells of IMR90 with/without Mito-Lyso linker. Nuclear DNA was labelled in blue with DAPI. Scale bar, 20 μm.

(K-M) Quantification of nuclear size (K), nucleolar size (L), and number of nucleolus (M) in pre-senescent and senescent cells of IMR90 with/without Mito-Lyso linker. More than 100 cells from three biological repeats were analyzed for each group.

(N, O) Representative images and size quantification of Golgi apparatus through immunofluorescence staining with GM130 antibody (Alexa Fluor® 647 conjugate) in IMR90 cells with/without Mito-Lyso linker. Nuclear DNA was labelled in blue with DAPI. Scale bar, 50 μm. The Golgi apparatus size is normalized to nuclear size. More than 100 cells from three biological repeats were analyzed.

Bar graphs are means ± SEM. Data were analyzed with unpaired two-tailed t test: *, $p<0.05$; **, $p<0.01$ ***, $p<0.001$; ****, $p<0.0001$; ns, not significant.

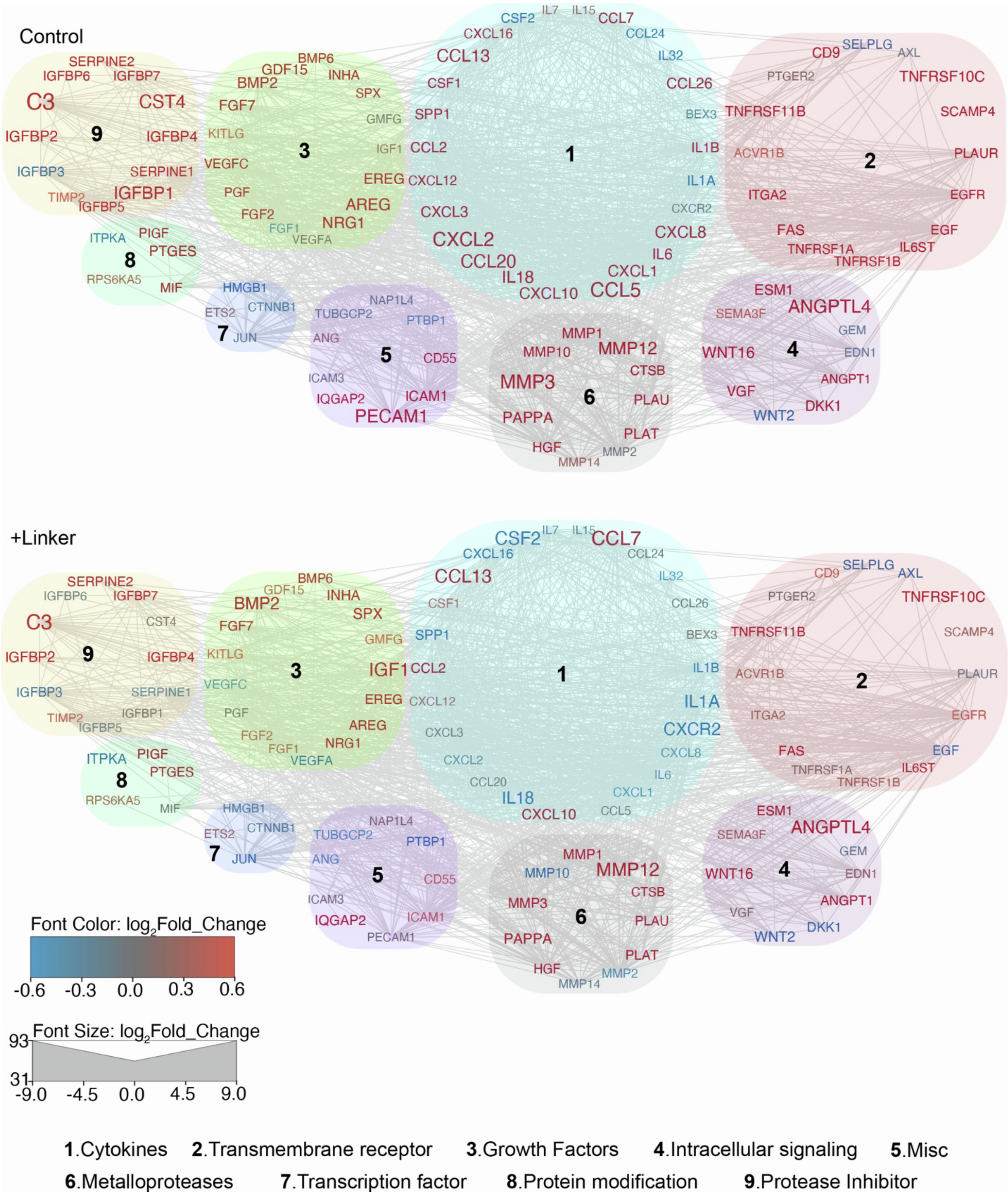

Figure S7. Comparison of the SenMayo network between control and linker-expressing senescent IMR90 cells. Related to Figure 7.

The SenMayo network consists of nine distinct protein classes that form a robust interaction network identified in prior studies. Each protein within the network is color-coded (red for upregulated and blue for downregulated) and its font size is scaled logarithmically ($\log_2$) based on expression changes relative to pre-senescent cells. Control, wild-type IMR90 cells. +Linker, Mito-Lyso linker-expressing IMR90 cells.
